# How is the brain affected by metabolically healthy or unhealthy obesity in adulthood and elderly? A narrative review of neuroimaging and neurocognitive findings

**DOI:** 10.3389/fnagi.2025.1616303

**Published:** 2025-12-01

**Authors:** Antonietta Pepe, Asieh Amousoltani Arani, Francesca Bracone, Augusto Di Castelnuovo, Loreto Muñoz-Venegas, Chiara Cerletti, Giovanni de Gaetano, Maria Benedetta Donati, Licia Iacoviello, Alessandro Gialluisi

**Affiliations:** 1 Department of Medicine and Surgery, LUM University, Casamassima, Italy; 2 Research Unit of Epidemiology and Prevention, IRCCS NEUROMED, Pozzilli, Italy

**Keywords:** metabolically healthy obesity, MRI brain imaging, neurodegenerative disease, neuropsychiatric disease, aging

## Abstract

The global aging of the population, coupled with an increasing prevalence of sedentary lifestyle and overnutrition, is fueling an alarming rise in the worldwide obesity rates. Besides its well-known bodily consequences, obesity is increasingly recognized as a risk factor for cognitive impairment, dementia, mood disorders, and emotional distress, suggesting a possible role of adiposity in the pathogenesis of both neurodegeneration and neuropsychiatric diseases. Despite the growing research interest, the pathophysiological mechanisms linking obesity to brain health remain poorly understood. Specifically, it is unclear whether the neuroanatomical, neurofunctional, and neurocognitive correlates of late-life obesity are directly imputable to either the excessive body fat accumulation or physiological age-related neurodegeneration, or if they are mediated by possible cardio-metabolic comorbidities which are common chronic conditions among the elderly. This narrative review synthesizes evidence on neuroimaging (MRI) and neurocognitive findings across adulthood and late life, with a focus on the metabolically healthy obese individuals, a sub-group of the obese population maintaining a favorable cardio-metabolic health profile. Direct studies on metabolically healthy obesity often report inconclusive evidence for the effect of obesity on neuroanatomical impairments or cognitive functions, and when the effects are present, they are much less pronounced compared to those observed in metabolically unhealthy individuals. Yet, many indirect studies reporting the effects of obesity after controlling for cardio-metabolic conditions suggest that obesity *per se* is associated with brain atrophy, reduced white matter integrity, and alterations in rewards-homeostatic-control networks. In conclusion, current evidence indicates that metabolically healthy obesity might not be entirely benign for brain health. More longitudinal multimodal imaging studies, with better characterization of both obesity and metabolic phenotypes, are therefore warranted to clarify trajectories and causal pathways.

## 1 Introduction

### 1.1 Background

The prevalence of excessive body weight is escalating at an alarming rate in all age groups, genders, ethnicities, and societies across the world (Phelps et al., 2024). In 2022, 2.5 billion adults (aged ≥ 18 years) were overweight (BMI ≥ 25 kg/m^2^), and about 670 million of these were obese (BMI ≥ 30 kg/m^2^), corresponding to approximately 13% of the global adult population and 31% of the US population aged ≥ 65 years (Malik et al., 2004; Phelps et al., 2024; United Health Foundation, 2024; WHO, 2000).

Obesity is one of the most important modifiable risk factors for premature mortality (Crotti et al., 2018; Ghulam et al., 2023) and is strongly associated with multiple health conditions, including insulin resistance, type II diabetes, cardiovascular disease, hypertension, dyslipidemia, osteoarthritis, chronic low-grade inflammation, and other complex comorbidities (Hruby et al., 2016; Kivimäki et al., 2022). In addition to its deleterious impact on quality of life, disability and morbidity rates of affected subjects, obesity has been increasingly recognized as a risk factor for neurological and neurodegenerative diseases. Growing evidence from epidemiologic and neuroimaging studies link obesity to accelerated brain aging (Debette et al., 2011; Gunstad et al., 2007; Whitmer et al., 2005), mild cognitive decline (Kharabian Masouleh et al., 2016), Parkinson’s disease (Kao et al., 2020), Alzheimer’s disease (Gustafson et al., 2003; Singh-Manoux et al., 2018; Tabassum et al., 2020; Whitmer et al., 2005), other dementias (Beydoun et al., 2008; Gustafson et al., 2003; Han et al., 2021; Lee et al., 2020, 2019; Monda et al., 2017; Pedditzi et al., 2016; Singh-Manoux et al., 2018; Whitmer et al., 2005) and ischemic stroke (Horn et al., 2021; Strazzullo et al., 2010). More specifically, neuroimaging evidence has highlighted a number of measurable effects of obesity onto the Central Nervous System, including reductions in total and regional gray matter through structural Magnetic Resonance Imaging (MRI) (García-García et al., 2019, 2022), microstructural alterations in white matter through diffusion tensor imaging (DTI) (Karlsson et al., 2013; Kullmann et al., 2016; Mueller et al., 2011; Papageorgiou et al., 2017), and altered activation and connectivity patterns in rewards- and executive-control networks via functional MRI (fMRI) (Zhang et al., 2020). Furthermore, previous neuropsychological studies have reported mild to moderate impaired cognitive performances in obese compared to normal weight subjects, especially in executive functions (Gunstad et al., 2007), such as episodic and working memory, processing speed, and attention (Dye et al., 2017; Kharabian Masouleh et al., 2016). Psychological well-being also appears to be compromised in obesity, with commonly reported symptoms including emotional distress (Steptoe and Frank, 2023), depression (Blasco et al., 2020), bipolar disorder (Kambey et al., 2023), as well as appetite dysregulation often involving binge-eating disorder (McCuen-Wurst et al., 2018).

Taken together, previous evidence indicates a possible involvement of excessive body fat in the pathogenesis of both neurodegeneration and neuropsychiatric diseases.

### 1.2 Challenges and aims

#### 1.2.1 Challenges

Given the aging of the worldwide population, the rising prevalence of both obesity and neurocognitive disturbances among older adults, and considering the economic and social burden of these conditions, unveiling the complex link between obesity and brain health in the mid- to late-life is of utmost importance. However, the pathophysiological pathways linking obesity to brain damage and/or impaired cognition are far from being fully understood, and a number of aspects remain to be clarified. Firstly, obesity is often comorbid with several cardio-metabolic abnormalities, namely hypertension, dyslipidemia, and poor glycemic control, the latter manifesting as either insulin resistance, impaired fasting glucose and/or tolerance, or manifest diabetes (García-García et al., 2022). Moreover, systemic inflammation is often characteristic of this condition (García-García et al., 2022). While neuroanatomical abnormalities in obese subjects with cardio-metabolic conditions have been documented using different brain MRI-derived biomarkers (Alfaro et al., 2018; Bokura et al., 2010; Yaffe et al., 2004), neuroimaging traits are notably under-investigated in metabolically healthy obesity (Angoff et al., 2022; Beyer et al., 2019b; Medic et al., 2016). Therefore, the neuroimaging literature is still inconclusive on whether patterns of aberrant structural/functional findings in brain MRI data are directly attributable to obesity itself, to physiological age-related neurodegeneration, or are possibly mediated through cardio-metabolic and inflammatory dysregulation (Beyer et al., 2019b; García-García et al., 2022). Secondly, conflicting findings have been reported in the literature, especially in the mid- to late- adulthood, on the association between obesity and cognitive disorders, with obesity being linked to both deleterious and protective effects on cognitive functions and dementia risk, a phenomenon often referred to as the “obesity paradox” (Buchman et al., 2005; Hughes et al., 2009; Pedditzi et al., 2016; Qizilbash et al., 2015; Xu et al., 2011).

Thirdly, the employed definition of metabolically healthy obesity and sample demographics typically differs among studies, thus preventing their direct comparison (Eckel et al., 2016).

#### 1.2.2 Aims

The purpose of this narrative review is to summarize the current brain MRI literature on metabolically (un)healthy adults with obesity, and to examine the link between obesity and cognitive impairment as assessed through test-based evaluations. Particularly, we aim to elucidate how obesity can coexist with apparently preserved metabolic health and cognitive function despite being widely recognized as a major risk factor for cardiovascular, metabolic, and neurodegenerative diseases. Furthermore, we attempt to shed light on the “obesity paradox” by clarifying whether obese individuals with preserved metabolic control are either at increased or reduced risk for impaired brain health and cognition as compared to their lean counterparts. In addition, we aim to highlight the biological mechanisms that possibly link obesity, its comorbidities, and brain health, with special attention to obesity-induced neuroinflammation and alterations in the blood–brain barrier. Recognizing these early indicators of neurodegeneration holds the potential to improve both disease diagnosis and treatment outcomes.

To reach these goals, we first define the metabolically healthy obese phenotype and describe the most common MRI-derived markers and neurocognitive traits tested for association with obesity (Section “2 Metabolically healthy obesity (MHO)”). We focus on evidence from structural MRI alterations typical of brain aging (including brain atrophy, vascular pathology, and loss of white matter integrity) and on functional MRI literature to examine how obesity relates to alterations in brain networks and task-related activation patterns. We then provide a narrative review of the neuroimaging findings in metabolically healthy obesity, linking them to neurocognitive findings, while speculating on the possible pathophysiological mechanisms underlying them (Section “3 Neuroimaging and neurocognitive findings in obesity and MHO”). Particularly, we highlight the role of inflammatory, hormonal and cerebrovascular mechanisms on the pathogenesis of impaired brain health as described in the neuroimaging and cognitive findings. Finally, we discuss current and future challenges in the field and the potential implications of the collected evidence for personalized public health strategies (Section “4 Discussions and conclusions”).

This review emphasizes that, although metabolically healthy obesity has been hypothesized to represent a relatively benign condition, it might still confer an increased risk for accelerated brain aging compared to metabolically healthy individuals with a healthy weight. This suggests a potential role of adipose tissue as an active endocrine organ damaging the central nervous system even in the absence of overt cardio-metabolic comorbidities. However, the available literature is sparse and partially contradictory, especially with respect to late adulthood, and more studies are needed to elucidate the complex interplay between obesity, metabolic health, brain health, and cognition.

## 2 Metabolically healthy obesity (MHO)

Obesity is a complex disease with multifactorial origin, including genetic, environmental, and lifestyle factors (De Lorenzo et al., 2019). Although it is commonly defined by a body mass index (BMI) ≥ 30 kg/m^2^ (BMI ≥ 25 kg/m^2^ for overweight status) (Malik et al., 2004), this metric is a simple measure of weight-to-height ratio that does not account for body composition such as muscle and fat mass or bone density. Alternative measures, such as waist circumference (WC), waist-to-hip ratio (WHR), and percentage of body fat (BF%) are more directly related to body fat distribution and central (abdominal) adiposity, and are generally recommended to complement BMI (De Lorenzo et al., 2019; Shen et al., 2023).

**FIGURE 1:**
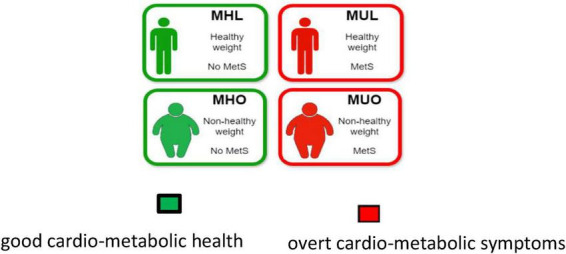
Stratification of adult population into four groups based on their combined obesity and metabolic status: metabolically healthy obese (MHO), metabolically unhealthy obese (MUO), metabolically healthy lean (MHL) and metabolically unhealthy lean (MUL) subjects.

The endophenotype of adult and elder obesity is extremely diverse from one subject to another and so are the health outcomes in both the central nervous system (CNS) and the peripheral nervous system (PNS). Indeed, the concept of metabolically healthy obesity has been introduced in clinical practice to describe the heterogeneity within the obesity population, particularly to differentiate the subgroup of individuals expressing a favorable metabolic profile (despite carrying extra body weight) from those who exhibit overt cardio-metabolic abnormalities (Blüher, 2020, 2010; Kouvari et al., 2023; Machado-Fragua et al., 2023; Mayoral et al., 2020). Following this, individuals might be stratified into four groups based on their combined obesity and metabolic status: metabolically healthy obese (MHO), metabolically unhealthy obese (MUO), metabolically healthy lean (MHL) and metabolically unhealthy lean (MUL) subjects (see Figure 1 and Box 1).

BOX 1 **MHO definition.**

As of today, there is no standardized definition for MHO and more than 30 different criteria have been proposed (Eckel et al., 2016; Gómez-Zorita et al., 2021; Ma et al., 2019; Primeau et al., 2011; Rey-López et al., 2014; Smith et al., 2011). Among the most used criteria in the neuroimaging literature are those proposed by the National Cholesterol Adult Treatment Panel III (NCEP-ATP-III) (Expert Panel on Detection, Evaluation, and Treatment of High Blood Cholesterol in Adults, 2001) and the International Diabetes Federation (IDF) (Mørkedal et al., 2014). The two differ in their definition of both “metabolically healthy” and “obese” within the MHO category: the IDF definition demands the absence of any metabolic disturbance but includes individuals in the overweight range (BMI ≥ 25 kg/m^2^ and/or WC ≥ 94 cm for men or ≥ 80 cm for women). In contrast, the NCEP-ATP-III criterion is less stringent for metabolic status, allowing individuals to have no more than one metabolic abnormality among dyslipidemia, hypertension and glycemic control, but includes only individuals with overt obesity (BMI ≥ 30 kg/m^2^ and/or WC: ≥102 cm for men or ≥88 cm for women). They also differ slightly in the definition of impaired glycemic control, as detailed in the following table (Table 2).

Notably, neither the NCEP-ATP-III nor the IDF criteria take into account traits of systemic inflammation, hormonal imbalance, or cardiorespiratory fitness status. Other MHO definitions, such as the one by Karelis et al. (2005) - based primarily on markers of insulin sensitivity, inflammation and lipid profile – and the one by Wildman (2008) - based on markers of the metabolic syndrome combined with the homeostasis model assessment of insulin resistance (HOMA-IR index) – exist, but have not been widely adopted in neuroimaging literature.

Recent meta-analyses have listed a number of physiological and phenotypic differences of MHO as compared to MUO individuals. Indeed, compared with MUO, MHO individuals are characterized by a milder degree of obesity, a more recent onset of overweight/obesity, and a more favorable fat distribution, characterized by greater subcutaneous rather than visceral or ectopic fat accumulation (Blüher, 2010; Eckel et al., 2016; Gómez-Zorita et al., 2021; Janssen et al., 2004; Ma et al., 2019; Primeau et al., 2011). They also demonstrate better hormonal control, higher insulin sensitivity, lower liver fat content, lower concentrations of hepatic enzymes, more favorable blood lipid profile, reduced atherosclerosis, less altered adipose tissue functions, smaller adipose cell sizes, and lower levels of systemic inflammatory markers. Lifestyle and functional traits also tend to be more favorable in MHO as compared to MUO individuals, including better cardiorespiratory fitness, higher engagement in physical activity, and better quality of sleep (Blüher, 2010; Eckel et al., 2016; Gómez-Zorita et al., 2021; Janssen et al., 2004; Ma et al., 2019; Primeau et al., 2011; Table 1).

**TABLE 1:** Endophenotypic traits in MHO (left) and MUO (right) individuals.

MHO	Trait/feature	MUO
Moderate	Fat mass	High
Subcutaneous	Main fat location	Visceral and ectopic
Low	Liver fat content	High
High	Insulin sensitivity	Low
Moderate	Triglycerides	High
Normal	Inflammatory markers	High
Low	HDL-c	High
High	Adiponectin	Low
Moderate	Fatty liver	Yes
Moderate	Cardio-metabolic risk	High
No or moderate	Hypertension	Yes
Hyperplasia	Adipocyte growth	Hypertrophy
Moderate	Cardiorespiratory fitness	Low
Moderate	Physical activity	Low
Women	Gender preference	Men

As compared to MUO, MHO individuals are characterized by a more favorable fat distribution, insulin sensitivity, systemic inflammation, cardiorespiratory fitness, and adipose tissue functions. Adapted from Blüher (2020), Gómez-Zorita et al. (2021), Primeau et al. (2011).

**TABLE 2:** Two commonly used definitions for the MHO phenotype.

MHO definition	2001 NCEP-ATP III criterion Obesity+ at most 1 trait among dyslipidemia, hypertension and glycemic control	IDF-2005 criterion (Funnell et al., 2017) Overweight/ obesity+ no other trait among dyslipidemia, hypertension and glycemic control
Unhealthy weight	BMI ≥ 30 kg/m^2^ WC: ≥102 cm (M) or ≥88 cm (F)	BMI ≥ 25 kg/m^2^ or WC: ≥94 cm (M) or ≥80 cm (F)
Dyslipidemia	HDL: <40 mg/dL (M) or <50 mg/dL (F) or TG ≥ 150 mg/dL or lipid-lowering medications	HDL: <40 mg/dL (M) or <50 mg/dL (F) or TG ≥ 150 mg/dL or lipid-lowering medications
Hypertension	SBP ≥ 130 mmHg or DBP ≥ 85 mmHg or anti-hypertension treatment	SBP ≥ 130 mmHg or DBP ≥ 85 mmHg or anti-hypertension treatment
Glycemic control	Fasting glucose ≥ 110 mg/dL or Glucose tolerance medication* Oral glucose tolerance - glycated hemoglobin Medical records of history/diagnosis of T2DM	Fasting glucose ≥ 100 mg/dL Anti-diabetic medications

*“Glucose tolerance medication” refers either to anti-diabetic medications or insulin. BMI, body mass index; DBP, diastolic blood pressure; HDL, high-density lipoprotein cholesterol; F, female; M, men; MHO, metabolically healthy obese; MUO, metabolically unhealthy obese; WC, waist circumference; SBP, systolic blood pressure; T2DM, type 2 diabetes mellitus; TG, triglycerides.

### 2.1 MRI-derived markers and cognitive scores for obesity research

Among various neuroanatomical imaging modalities, MRI is a non-invasive *in vivo* examination tool that has attracted a substantial share of interest in research and clinical practice due to its wide availability, good spatial resolution, and absence of radiation exposure. There are several structural brain MRI techniques that have been used in obesity studies to investigate neuroanatomical changes (Medic et al., 2016), essentially via biomarkers of gray matter (GM) and white matter (WM) integrity. Beyond structural MRI, a growing number of studies has employed functional MRI which provides an indirect assessment of brain-activity through blood-oxygen-level-dependent (BOLD) signals, reflecting local changes in cerebral blood flow and oxygen metabolism. A schematic overview of these imaging modalities and derived measures is provided in Boxes 2, 3 and Tables 3, 4, while a more detailed description is provided in the Supplementary material. Other MRI sequences and MRI-derived markers of brain pathology have been reviewed elsewhere (Tang et al., 2021) and are not presented here.

BOX 2 **Brain structural MRI modalities and derived metrics (see also Supplementary material).**

Structural MRI

T1-weighted (T1-w) head MRI modality is sensitive to the signal of the fatty tissue and provides good contrast between gray (GM), white matter (WM) and cerebrospinal fluid (CSF). Once T1-w MRI images are segmented, total brain volume can be estimated. Among the most used global volumetrics derived from T1-w MRI imaging are the Total Cerebral Volume (TCV), GM volume and WM volume. Particularly, TCV is an established macroscopic markers of brain atrophy, neurodegeneration (Moran et al., 2017), and brain aging (Tsao et al., 2013). Aside from global volumetric metrics, T1-w MRI brain can be analyzed via spatially fine-grained metrics using volumetric or surface-based approaches. Voxel-based morphometry (VBM) can be used to quantify macroscopic abnormalities in GM and WM composition and can reveal brain atrophy. Surface Based Morphometry (SBM) can be used to derive morphometric measures from GM cortex. Of particular clinical relevance is cortical thinning which indicates possible axonal loss, reduced size of neural cell bodies, and/or demyelination. This has been associated to aging (Salat et al., 2004), declined executive functions (Burzynska et al., 2012) and impaired intelligence (Schnack et al., 2015). Cortical surface area (CSA), on the other hand, has been hypothesized to mirror the tension between deep WM fibers.

T2-weighted (T2-w), Proton Density-weighted (PD), and Fluid-Attenuated Inversion Recovery (FLAIR) MRI data can be used to detect macroscopic areas of WM pathology associated to aging-related processes and cerebrovascular damage. White Matter Hyperintensities (WMH), lacunes, microbleeds (they require sequences sensitive to susceptibility, typically T2*-w and SWI), and enlarged perivascular spaces are well-established markers of cerebral small vessel disease (cSVD) (Duering et al., 2023). WMH prevalence increases with age, obesity, hypertension, diabetes, but also with unhealthy lifestyles such as smoking and sedentary life (Hakim, 2019; Wardlaw et al., 2015). WMH are clinically relevant as they highlight microvascular lesions in the cerebral white matter, possibly resulting from demyelination and axonal loss, and have been linked with an increased risk of stroke, cognitive impairment, dementias, neurological diseases, and late-onset depression (Debette and Markus, 2010; Herrmann et al., 2007; Kalaria et al., 2012; Lampe et al., 2019; Tosto et al., 2014). Moreover, WMH can be used as an anatomical signature of cognitive decline and dementia (Marseglia et al., 2019). Other cSVD markers have also been previously associated with cognitive impairment, including lacunes (Makin et al., 2013), microbleeds (Cordonnier et al., 2007; Vermeer et al., 2007; Shenton et al., 2012).

Diffusion Weighted Imaging (DWI) -Diffusion Tensor Imaging (DTI) is a type of DWI- is an MRI modality especially suited to identify the presence and location of microstructural WM lesions, even at the early stages of neuropathology (Tang et al., 2021). By characterizing the diffusion properties of water molecules in the white matter fibers, it is possible to detect microstructural abnormalities and compromised WM integrity, possibly resulting from axonal injuries. It can also be used to estimate structural connectivity (Shenton et al., 2012).

BOX 3 **Brain functional MRI modalities and derived metrics.**

Functional MRI

Resting-state functional MRI (rs-fMRI) assess spontaneous neuronal activity by measuring low-frequency (<1 Hz) fluctuations in Blood-Oxygenation-Level-Dependent (BOLD) signals on individuals who are not engaging in a specific task (at rest) (Wang et al., 2025). The rs-fMRI signals measure local changes in cerebral blood flow and oxygenation, which are assumed to reflect the intrinsic functional interaction between brain regions. Among the most used rs-fMRI-based metrics is the Seed-Based Connectivity (SC) which quantifies temporal correlations between predefined regions of interest -typically large-scale networks such as the default mode network (DMN), salience network (SN), and executive control network (ECN)- and the rest of the brain. Abnormalities in SC connectivity are linked to dysregulated self-referential processing and salience attribution, which are relevant for eating behavior. In addition, independent component analysis (ICA) allows for the identification of spatially independent networks, providing insights into intrinsic brain organization without requiring *a priori* hypotheses. Also, the measures the intensity of spontaneous neural activity in specific brain regions, serving as proxies for regional spontaneous brain activity. Correlation patterns among large-scale networks in spatially remote areas, assessed via Functional Connectivity (FC), have been also used to study the neuronal control of food intake (Lips et al., 2014). Finally, Regional Homogeneity (ReHo) measure assesses local synchronization of BOLD signals, reflecting the functional coherence of neighboring voxels.

Task-based functional MRI (task-fMRI) measures task-evoked changes in blood oxygenation (BOLD) signal to identify brain regions engaged during controlled stimuli or cognitive activities (Poldrack, 2007). In obesity research, experimental paradigms usually pertain food-cue reactivity, rewards valuation, and executive control tasks to probe specific neural systems underlying motivation, self-regulation, and rewards processing (Stoeckel et al., 2008; García-García et al., 2013). Fluctuations of the BOLD signals are interpreted as indirect markers of local neuronal activation mediated by neurovascular coupling (Logothetis et al., 2001). Task-fMRI data typically undergo a standard preprocessing pipeline, including motion correction, spatial normalization, temporal filtering, and smoothing, followed by first-level general linear modeling (GLM) to estimate voxel-wise task-related responses (Friston et al., 1994). A common derived metric is task-evoked activation contrasts to identify brain regions that respond selectively to specific conditions (e.g., food vs. neutral stimuli). Psychophysiological interaction (PPI) analyses assess task-modulated functional connectivity between brain regions, shedding light on how cognitive or emotional demands alter network interactions (Friston et al., 1997). Multivoxel pattern analysis (MVPA) and decoding approaches evaluate distributed patterns of activation, providing insights into representational coding of stimuli such as food cues or rewards signals.

**TABLE 3:** Summary of the main structural MRI-derived markers to assess brain pathology.

MRI	Method	Metric	Definition	Biological phenomenon/clinical significance
T1-w	Volumetric approach	Total cerebral volume (TCV)	Total volume enclosed by the outer surface of the brain.	↓ Associated with brain atrophy and neurodegeneration due to aging or disease.
		GM volume (GMV)	Volume of GM tissues.	↓ Associated with GM atrophy and neurodegeneration due to aging or disease.
		WM volume (WMV)	Volume of WM tissues.	↓ Associated with demyelination, axonal loss and other WM degeneration due to aging or disease.
T1-w	Voxel-based morphometry (VBM)	GM composition	Voxel-level GM volume (or density*).	↓ Associated with GM atrophy and neurodegeneration due to aging or pathology.
		WM composition	Voxel-level WM volume (or density*).	↓ Due to aging or pathology.
		CSF composition	Voxel-level CSF volume (or density*).	↑ Due to vessel enlargement and brain tissue shrinkage.
T1-w	Surface-based morphometry (SBM)	Cortical thickness (CT)	Vertex-level distance between the inner and outer surface of the brain.	↓ (thinning) Associated with cortical atrophy due to aging or pathology. Focal patterns of CT ↑ (thickening) might be due to brain pathology.
		Cortical surface area (CSA)	Vertex-level area of the outer surface of the brain.	CSA is typically anticorrelated to CT. CSA possibly mirrors the tension between deep WM fibers.
		Cortical volume (CV)	Vertex level volume of the cortical ribbon (enclosed by the inner and outer surfaces of the brain). CV = CT × CSA.	See Supplementary materials
T2-w, SD, FLAIR		White matter hyperintensities (WMH)	T2 signal hyperintensity of variable size, quantified with grading on visual rating scale or total volume based on image segmentation.	↑ Increase indicative of macroscopic WM damage due to cSVD. Associated with greater age, increased risk of stroke, cognitive impairment, dementia, and death (especially due to cardiovascular causes) (Kim et al., 2017).
		Lacunes	Fluid-filled cavities up to 15 mm diameter, often with hyperintense rim on FLAIR, quantified by counting.	↑ Macroscopic WM damage due to aging, cSVD, may result from a vascular insult or hemorrhages. Associated with increased risk of cognitive impairment, dementia, stroke, and mortality (Kim et al., 2017; Makin et al., 2013).
		Cerebral microbleeds	Small areas of signal void on T2*-w or other sequence sensitive to susceptibility, quantified by counting.	↑ Microscopic bleeding due to aging, cSVD. Linked to increased risk of ischemic and hemorrhagic stroke, cognitive decline, dementia, AD, inflammatory status (Yates et al., 2014).
		Perivascular spaces	Fluid-filled space following the typical course of a vessel penetrating the brain parenchyma, quantified with grading on visual rating scale or volume/number quantification based on image segmentation.	↑ Enlargement of perivascular spaces indicative of aging, cSVD, arterial stiffening, excessive protein accumulation in vessels. Commonly associated with cognitive decline, dementia, AD, inflammatory status (Bown et al., 2022; MacLullich et al., 2004).
DW	DTI/Free-water DTI modeling	Fractional anisotropy (FA)	Metric of directionality of the water diffusion in the diffusion tensor model.	↓Decrease in case of unrestricted diffusion of water molecules, indicative of microscopic WM damage (axon demyelination) due to aging or pathology, also associated with memory and learning deficits.
		Mean diffusivity (MD)	The mean diffusivity in each of the three principal orientations in the diffusion tensor.	↑Increase indicative of axon demyelination, inflammation, or increased tissue water content due to aging or pathology.
		Free Water (FW)	Water molecules that are not restricted or directed and thus represents the extracellular space.	↑ Increase indicative of microscopic loss in WM integrity due to damage to the axonal structure and/or myelin membrane surrounding WM fibers. It has been linked to aging and pathology, including early stages of cSVD.

*Composition refers to brain tissue’ density or volume, depending if images have been modulated or not (see Supplementary material).

**TABLE 4:** Summary of the main functional MRI-derived markers to identify changes in baseline functional architecture of intrinsic networks (rs-fMRI) or changes in the way these networks are dynamically engaged during specific tasks (task-fMRI).

fMRI	Metric	Definition	Biological phenomenon/clinical significance
Resting-state	Functional connectivity (FC)	Temporal correlation of spontaneous BOLD signals between large-scale networks in spatially remote areas at rest.	Abnormal FC can identify patterns of altered network integrity. Altered FC has been reported in obesity within rewards-related (striatum, orbitofrontal cortex), salience, and DMN networks, and linked to dysregulated appetite control and rewards sensitivity (Kullmann et al., 2012; Lips et al., 2014; García-García et al., 2019; Schwartz et al., 2000).
	Seed-based connectivity (SC)	Functional connectivity (FC) between a predefined “seed” region and other brain regions.	Reduced (↓) SC can identify targeted network disruptions. In particular, reduced SC in prefrontal control over striatal regions has been associated with food craving and impulse dysregulation (García-García et al., 2013; Lips et al., 2014).
	Independent component analysis (ICA)	Decomposition of BOLD signals into independent spatial networks.	Used to identify rs networks (DMN, salience, executive control) and assess obesity-related reorganization or reduced network segregation.
	Regional homogeneity (ReHo)	Similarity or synchronization of BOLD time series between neighboring voxels.	Indicates local neural coherence. Decreased ReHo in prefrontal and parietal areas has been observed in obesity and may relate to impaired executive functions and impaired inhibitory control.
Task	Task-evoked activation	Identifies brain regions showing significant task-related BOLD signal changes, typically contrasting experimental conditions (e.g., food vs. neutral cues).	Reveals hyperactivation in rewards-related (striatum, orbitofrontal cortex, amygdala) and homeostatic regions during food-cue exposure; hypoactivation in prefrontal control areas linked to impaired self-regulation (Stoeckel et al., 2008; García-García et al., 2013; Rothemund et al., 2007).
	Psychophysiological interaction (PPI)	Examines task-modulated connectivity between a seed region and other brain areas.	Reveals altered coupling between prefrontal control and limbic/rewards circuits (e.g., diminished top-down control in obesity) (Friston et al., 1994; Kullmann et al., 2012).

BOLD, blood-oxygenation-level-dependent; DNN, default mode network; FC, functional connectivity; FA, fractional anisotropy; fMRI, functional magnetic resonance imaging; ICA, independent component analysis; MRI, magnetic resonance imaging; PPI, psychophysiological interaction; ReHo, regional homogeneity; rs, resting-state; SC, seed-based connectivity; SN, salience network.

In the context of evaluating cognitive dysfunctions in MHO, a number of neurocognitive tests are typically administered to quantify global cognitive performances and executive functions, particularly in the domains of episodic and working memory, processing speed, attentional control, and response inhibition functions (Gunstad et al., 2007). Executive functions play a crucial role in influencing dietary intake and eating choices – and in turn maintaining equal or negative energy balance –through regulation of impulse control, self-monitoring, and goal-directed behavior (Gonçalves et al., 2014; Gunstad et al., 2007; Wyckoff et al., 2017). A summary of these scales, which are also widely used for monitoring age-related cognitive decline, is provided in Table 5.

**TABLE 5:** Summary of the main neurocognitive tests used to assess different cognitive domains.

Cognitive function	Test	Description/utility	Cognitive domains assessed
Global cognitive function	MMSE (Mini-Mental State Examination) (Folstein et al., 1975).	A brief (30-points) questionnaire commonly used to screen for cognitive impairment.	Orientation, recall, attention, calculation, language, visual-spatial skills.
	MoCA (Montreal cognitive assessment) (Nasreddine et al., 2005).	A 30 points scale - more sensitive than MMSE - for detecting mild cognitive impairment. All adult populations, not limited to the aging population.	Attention and concentration, executive functions, memory, language, visuoconstructive abilities, abstraction, calculation, orientation.
	Blessed information-memory-concentration test (BIMC) (Kawas et al., 1995).	A brief screening tool (28-points) consisting of 6 items. Commonly used in elderly populations to assess cognitive decline and dementia.	Memory, concentration, orientation, attention.
Attention and executive functions	Digit span (forward and backward) (Ostrosky-Solís and Lozano, 2006).	A subtest of the Wechsler Adult Intelligence Scale (WAIS), consisting of two parts: digit span forward [repeat a series of digits in the same order (9- points)] and digit span backward [repeat a series of digits in reverse order (9- points)].	Attention, concentration, working memory.
	Trail making test (TMT) A and B (Carone et al., 2007).	A neuropsychological test consisting of two parts: TMT A requires connecting numbered circles in sequential order, while TMT B involves alternating between numbers and letters. The score reflects completion time.	Visual attention, task switching, cognitive flexibility.
	Stroop test (Periáñez et al., 2021).	A psychological test consisting of three parts: reading color words, naming the ink colors of color words, and an incongruent condition where the ink color and the word meaning differ. Performance based on reaction time and accuracy; no traditional scoring system based on points.	Cognitive control, processing speed, inhibitory control.
	Wisconsin card sorting test (WCST) (Heaton et al., 1993).	A neuropsychological test (128-points) consisting of 128 cards that vary in color, shape, and number. Participants must infer the new rule based on feedback (“correct” or “incorrect”).	Executive function, including flexibility in thinking, problem-solving.
	Digit symbol substitution test (DSST) (Jaeger, 2018).	A neuropsychological test (93 points) consisting of 93 pairs of symbols and digits. The total score is based on the number of correct matches made in 90 s.	Processing speed, attention, psychomotor performance.
Memory	Verbal learning test (VLT) (Delis et al., 2010).	A cognitive assessment consisting of 15 words presented over multiple trials (15-points). Participants are asked to recall the words immediately after the presentation and again after a delay.	Verbal memory, learning ability, recall.
	Benton visual retention test (BVRT) (Benton, 1974).	A visual memory test (10-points) consisting of 10 designs that are presented for a brief period.	Visual memory, perception, visual-spatial abilities.
	Prospective memory test (PM) (Einstein and McDaniel, 1990).	A cognitive assessment (12-points) designed to evaluate the ability to remember to perform actions in the future.	Prospective memory, planning, self-initiation.
Language	Letter and category fluency (Gladsjo et al., 1999).	A cognitive assessment (20 points) that includes two parts: letter and category fluency. Participants are asked to generate as many words as possible beginning with a given letter or within a semantic category.	Verbal fluency, executive function, language processing.
	Boston naming test (Kaplan et al., 2016).	A cognitive assessment (60-points) consisting of 60 pictures of objects that participants must name.	Confrontational naming, language processing, lexical retrieval.
Visuospatial skills	Card rotations test (Wilson et al., 1980).	A visuospatial test (20 points) consisting of 20 cards depicting various shapes at different orientations. Participants must identify the correct orientation of each shape.	Visuospatial skills, mental rotation, spatial visualization

## 3 Neuroimaging and neurocognitive findings in obesity and MHO

Most of the existing neuroimaging and neurocognitive literature has investigated the deleterious impact of single traits of the metabolic syndrome separately –including obesity, impaired glucose metabolism, hypertension, dyslipidemia, and systemic inflammation– rather than considering their combined effects (Bahchevanov et al., 2021; Yaffe et al., 2004). As an example, obese individuals with type 2 diabetes have been reported to suffer from cognitive decline (Yoon et al., 2017), reduced total and regional brain volume (West et al., 2020), greater burden of WM hyperintensities (van Bloemendaal et al., 2016), disrupted resting-state functional connectivity in memory and executive functions (Cheke et al., 2017; Gao et al., 2025), and abnormal activation in task-fMRI paradigms including food cues and memory tasks (Guzzardi and Iozzo, 2019; Meng et al., 2020). Indeed, a recent study showed that obesity and metabolic health have an additive effect on cognitive dysfunction (Lyall et al., 2016). However, the impact of being overweight or obese in the absence (or presence of only a single) cardio-metabolic disturbance (i.e., MHO) on cognition and brain health is less well understood, particularly in the adult lifespan (García-García et al., 2022). While the MHO phenotype has often been regarded as a relatively benign status (Primeau et al., 2011), it has been hypothesized that it might still confer an increased risk of neurodegeneration and neurocognitive disturbances compared with MHL individuals.

Nonetheless, there is conflicting evidence for this hypothesis. On one hand, a number of studies in mid-life and elderly MHO subjects have reported an increased risk of cardiovascular events relative to MHL subjects, albeit to a lesser degree than in metabolically unhealthy subjects (Kramer et al., 2013; Lavie et al., 2018). Similarly, high BMI has been associated with MRI evidence of increased brain atrophy and diminished WM integrity, consistent with the hypothesis of accelerated brain aging [reviewed in García-García et al. (2022)]. On the other hand, epidemiological studies on large-scale cohorts have reported that, compared to MHL subjects, MHO individuals show a decreased risk of death, cardiovascular disease, cognitive decline, AD and other dementias (Cho J. et al., 2021; Cho Y. K. et al., 2021; Lee et al., 2019; Ma et al., 2019). This paradoxical evidence is corroborated by a neuroimaging study in the elderly reporting higher whole brain and hippocampal volume in MHO compared to MHL, alongside a better clearance of (see Section 3.3.1 below). These counterintuitive findings are most commonly reported in older samples and are referred to as the “obesity paradox” (Lee et al., 2019; Ma et al., 2019, 2018).

To attempt to clarify these inconsistencies, we review the relevant brain MRI and neurocognitive literature, with a focus on mid- to late-adulthood. We summarize findings from both structural (T1-w, T1-w, T2-w, SD, FLAIR, DW) and functional (rs-fMRI and task-fMRI) MRI modalities. We first review studies reporting the impact of obesity onto brain health not taking into account the metabolic health status (Section “3.2 Obesity findings”). Next, we collect evidence on the MHO phenotype from the relatively few “direct studies,” namely those comparing directly the MHO phenotype to either the MUO or MHL groups (Section “3.3.1 Direct studies”). Finally, we review the brain MRI literature for “indirect studies” on adult obesity controlled for metabolic health, namely studies investigating the effects of obesity (either dichotomic or continuous indices) stratified by metabolic control (mainly glucose tolerance and/or insulin resistance, cholesterol and blood pressure), or controlling for possible obesity-related comorbidities as confounders (Section “3.3.2 Indirect studies”).

### 3.1 Inclusion/exclusion criteria

We searched PubMed and Web of Science using combinations of keywords related to “obesity,” “metabolic health,” “neuroimaging,” “MRI,” “fMRI,” “cognition,” and “brain function.” Eligible studies included peer-reviewed articles on adult human populations (≥18 years) that applied MRI-based techniques (structural, diffusion, or functional MRI) and neurocognitive testing to investigate brain outcomes in individuals with overweight or obesity, with or without consideration of metabolic health status. More specifically, for the “direct studies,” we only reviewed those studies where the sample was stratified by both obesity status and metabolic control following one of the definitions in Table 2. For the “indirect studies,” we only considered studies with complete information on obesity status, with a sound control for the metabolic panel.

Only studies including full-head structural (T1-w, T2-w, SD, FLAIR, DW, see Table 3) or functional (rs-fMRI and/or task-fMRI, see Table 4) MRI data, with or without accompanying neuropshycometric tests, were reviewed. Only in case of “direct studies,” given the scarcity of existing literature, we also included studies with no MRI imaging data but with at least a neuropshycometric test on global or domain-specific (attention and executive functions, memory, language, and/or visuospatial skills) cognitive functions (see Table 5).

Exclusion criteria were applied to animal studies, pediatric samples (<18 years), articles not written in English, conference abstracts, and case report studies. Additionally, we excluded studies involving subjects affected by major neurological disorders (stroke, clinical dementia, neurodegenerative disease), major psychiatric illness (schizophrenia, bipolar disorder), current substance dependence, bariatric surgery, and pregnancy. Additional exclusions applied when sample size was <20.

### 3.2 Obesity findings

The MRI literature has highlighted several neuroanatomical changes in obese compared to normal weight individuals. Among the most consistently reported alterations is the reduced global GM volume (Bobb et al., 2014; Han et al., 2021; Janowitz et al., 2015; Raji et al., 2010), in line with large-scale evidence of its inverse association with BMI (Hamer and Batty, 2019), suggesting that obesity may be associated with accelerated brain atrophy. Besides evidence of global brain shrinkage, patterns of regional GM volume reductions detected through VBM have also been reported in obese adults, most prominently in prefrontal regions like the orbitofrontal cortex (OFC), inferior frontal gyrus (IFG), and medial prefrontal cortex (PFC) (García-García et al., 2019; Kharabian Masouleh et al., 2016; Kurth et al., 2013; Opel et al., 2015; Pannacciulli et al., 2006; Raji et al., 2010; Shott et al., 2015; Taki et al., 2008; Walther et al., 2010). Reduced GM volume in prefrontal regions may result from multiple additive mechanisms – including arterial stiffness, hypertension, and microvascular damage – which can lead to chronic hypoperfusion, thereby compromising neuronal integrity. These mechanisms may particularly affect the prefrontal cortex due to its high metabolic demand and sensitivity to oxygen and glucose deprivation (Gorelick et al., 2011; Willette and Kapogiannis, 2015). These results are also confirmed in studies reporting patterns of cortical thickness (CT) shrinkage in association with obesity of the prefrontal cortex - such as in the OFC (Medic et al., 2016), the ventromedial PFC, and the anterior cingulate (Marqués-Iturria et al., 2013) - so much so that prefrontal brain structural alterations have been hypothesized to mediate the genetic risk for obesity (Opel et al., 2021). The ventromedial PFC plays a key role in decision-making, executive control, rewards processing, and impulse regulation (Grafman and Litvan, 1999), and has been suggested as a possible neurobiological underpinning of obesity (Cieslik et al., 2015; García-García et al., 2019). More particularly, the ventromedial PFC mediates “hot” executive functions, namely cognitive functions involving rewards or affective-related domains, mostly related to tasks such as obeying to social rules, the interpretation of complex emotions, and the inhibition of inappropriate responses such as overeating behaviors (Grafman and Litvan, 1999). The medial PFC (particularly its ventral subdivision) has therefore been suggested as a neurobiological link between self-regulatory deficits and defective regulation of appetite and eating behaviors (García-García et al., 2019). However, the literature is not always concordant with this hypothesis. As an example, the OFC and the whole medial PFC has been reported to be enlarged (Horstmann et al., 2011; Weise et al., 2017), reduced (García-García et al., 2019; Marqués-Iturria et al., 2013; Medic et al., 2016), or not significantly affected (Kullmann et al., 2012; Sharkey et al., 2015; Zhang et al., 2017) in obesity. Some neuroanatomical findings, such as cortical thinning in the PFC, have been more consistently reported in the elderly than in younger adults, suggesting it might be a phenomenon occurring later in life or possibly secondary to obesity (García-García et al., 2019). Beyond structural alterations, multiple rs-fMRI studies in obese individuals have reported altered intrinsic activity and functional connectivity in networks underlying rewards-related regions (striatum, orbitofrontal cortex, amygdala), the salience network (insula, striatum), and executive/attentional control networks (prefrontal cortex) (García-García et al., 2015, 2019; Kullmann et al., 2023; Zhang et al., 2020). These systems are critical for self-regulation, rewards processing, and homeostatic control of appetite (Zhang et al., 2020; García-García et al., 2013; Kullmann et al., 2012; Lips et al., 2014). Such alterations may help explain overeating behaviors, including food cravings, disinhibition, “food addiction,” impulsivity, and the difficulty many individuals face in sustaining weight loss interventions (Kullmann et al., 2023; Li et al., 2023; Hogenkamp et al., 2016; Park et al., 2015).

Besides PFC, reduced GM has also been reported in hypothalamic regions (Kurth et al., 2013). The hypothalamus plays a key role in regulating food intake and energy expenditure (Velloso, 2009), specifically in mediating hunger versus satiety feelings, and pro-versus anti-thermogenesis in response to the amount of energy stored (Flier and Maratos-Flier, 1998; Schwartz et al., 2000; Velloso, 2009). In addition, evidence from rs-fMRI data indicates that obese individuals in a fasting state have stronger functional connectivity (FC) between brain areas involved in cognitive control, motivation, and rewards (e.g., medial PFC and dorsal striatum) as compared to lean subjects, suggesting an hypersensitivity to food cue in a fasting state (Lips et al., 2014). Other studies have reported evidence of brain atrophy also in areas involved in motor functions and emotions, such as the cerebellum and hippocampus (García-García et al., 2019; Han et al., 2021; Kurth et al., 2013; Medic et al., 2016; Opel et al., 2021, 2015; Walther et al., 2010). Notably, studies have reported altered functional connectivity between salience and hypothalamic networks in obesity, suggesting an imbalance between rewards sensitivity and self-regulation, resulting in disruption of rewards–homeostatic control circuits (Lips et al., 2014; Kullmann et al., 2012; García-García et al., 2013, 2014). Reduced connectivity within homeostatic and interoceptive regions (insula, hypothalamus) has also been described as a hallmark of the obese brain, potentially impairing satiety signaling.

These findings are complemented by task-fMRI evidence. Studies using food-cue paradigms consistently report hyperactivation of rewards-related regions (ventral striatum, orbitofrontal cortex) in obese compared to lean individuals (Rothemund et al., 2007; Stice et al., 2008), alongside hypoactivation in cognitive control regions (dorsolateral prefrontal cortex) during inhibitory control tasks, reflecting a reduced ability to resist temptation (Bruce et al., 2010).

Indeed, evidence from human studies shows that obesity and high-fat/high-cholesterol diets can disrupt blood-brain-barrier integrity, increase its permeability, and facilitate the entry of inflammatory molecules into the brain. This phenomenon has been consistently demonstrated in experimental studies on high-fat/high-cholesterol fed rodents, and related more specifically to hippocampal dysfunctions (Freeman and Granholm, 2012; Kanoski et al., 2010; Kanoski and Davidson, 2011) and to impaired clearance of amyloid-β, a peptide whose accumulation is central to Alzheimer’s pathology (Abdallah et al., 2021; Keaney et al., 2015; Ma et al., 2018). These mechanisms may accelerate neuroinflammation, neurotoxicity, and neurodegeneration, providing a plausible biological link between peripheral metabolic disturbances (such as obesity, insulin resistance and systemic inflammation) and central nervous system damage and thus cognitive decline.

Regarding WM damage, increased markers of cerebral small vessel disease (cSVD), such as white matter hyperintensities (WMH) load (Caunca et al., 2019a,b; Debette et al., 2011; Haltia et al., 2007; Kim et al., 2017), cerebral microbleeds (Kwon et al., 2016), and lacunar infarcts (Kim et al., 2017; Winter et al., 2008) have been reported in obese individuals free from cognitive impairment and neurological conditions. These findings are corroborated by other reports of microstructural white matter damage, as quantified by reduced integrity from Diffusion Weighted Imaging (DWI) or Diffusion Tensor Imaging (DTI) data in distributed tracts, including the corpus callosum, cingulum, cerebellar peduncles, and corona radiata (Karlsson et al., 2013; Kullmann et al., 2016; Mueller et al., 2011; Papageorgiou et al., 2017). Similar alterations were found in the internal and external capsule, the inferior longitudinal fasciculus, and the inferior occipitofrontal fasciculus – see (García-García et al., 2019) for a review. Taken together, these diffuse patterns of WM damage might accumulate in individuals with obesity and lead to a premature loss of brain tissue (atrophy) and affect cognitive performance.

Regarding neurocognitive literature, mid-life obesity has been linked to impaired cognitive domains, including episodic memory, working memory, executive functions, and decision-making capabilities, which might contribute to poor dietary choices (Dye et al., 2017). However, performances in domains such as executive functions and memory – which are both progressively impaired in physiological aging – have been reported to be altered in non-demented individuals with obesity in some studies (Gunstad et al., 2007) but not in others (Whitmer et al., 2005). These discrepancies were partially dependent on age, gender, and fitness status of the studied sample, and/or on the incomplete confounding adjustment (García-García et al., 2019; Kharabian Masouleh et al., 2016; Prickett et al., 2015), as also reported in Section “4.1 Methodological limitations in the literature.”

### 3.3 MHO findings

#### 3.3.1 Direct studies

The MRI literature of neuroanatomical changes in metabolically healthy obesity, especially in late adulthood, is relatively scarce, see Table 6. Among the few direct brain MRI studies (Angoff et al., 2022; Nam et al., 2020; Ma et al., 2019), the differences in the definition of MHO and sample selection complicate direct comparisons among studies (García-García et al., 2022; Eckel et al., 2016).

**TABLE 6:** Summary of (structural and functional) neuroimaging and neurocognitive findings in MHO direct studies.

Study (year)	Sample size/ population	Definition of MHO	Imaging modality	Cross-sectional/ longitudinal	MHO vs. MHL	MHO vs. MUO	Main conclusion
Angoff et al., 2022	2,170 young to middle-aged adults (Framingham Heart Study)	NCEP-ATP III criteria	Structural MRI (T1-w + DTI) + neurocognitive test	Cross-sectional	MHO showed intermediate brain aging compared to MHL; impaired global cognitive score, verbal memory, and abstract reasoning; higher FW content and lower FA	MUO exhibited accelerated brain aging lower total cerebral volume	Metabolic health moderates obesity-related GM loss
Nam et al., 2020	3165, age 50–69, neurologically healthy participants	HOMA-IR and lipid profile	Structural MRI: Silent brain infarcts (SBI)	Cross-sectional	MHO not significantly different from MHL in SBI prevalence	MUO associated with increased SBI risk	WM microstructure better preserved in MHO
Ma et al., 2019	6,151 Chinese elders	IDF criteria	Longitudinal imaging follow-up + cognitive assessment	Cross-sectional	MHO had lower risk of AD compared to MHL	MUO showed increased AD risk	MHO phenotype shows preserved brain structure
Kouvari et al., 2023	1,772 middle-aged and older adults (Framingham Offspring Study)	NCEP-ATP III + inflammatory markers	Cognitive assessment	Longitudinal	Stable MHO had comparable cognition to MHL	Transition from MHO → MUO linked to cognitive decline	Cognitive advantage for MHO compared to MUO
Dunn et al., 2023	78 middle-aged adults	Obesity (BMI) + insulin resistance (HOMA2)	Task-fMRI	Cross- sectional	MHO had a reduced striatal response to palatable taste as compared to MHL	MHO had an increased striatal response to palatable taste as compared to MHL	MHO responses are intermediate between MHL and MUO

Notably, neither the NCEP-ATP-III nor the IDF criteria take into account traits of systemic inflammation, hormonal imbalance, or cardiorespiratory fitness status. Other MHO definitions, such as the one by Karelis et al. (2005) - based primarily on markers of insulin sensitivity, inflammation and lipid profile - and the one by Wildman (2008) - based on markers of the metabolic syndrome combined with the homeostasis model assessment of insulin resistance (HOMA-IR index) - exist, but have not been widely adopted in neuroimaging literature.

In Angoff et al. (2022), associations of metabolic health and obesity with brain health were investigated in a direct study of early- to middle-aged adults free from prevalent diabetes or brain disorder. The sample consisted of 2170 individuals from the Framingham Heart Study Exam 2 cohort (mean age: 46 ± 9 years, 54% women, MHO/MUO/MHL/MUL: 423/198/1,385/164, predominantly Western European) and included T1-weighted and DTI along with neurocognitive data spanning executive function, memory, abstract reasoning, and visual processing. The MHO group was defined using the National Cholesterol Education Program Adult treatment panel III (NCEP-ATP III) criterion, as in Ma et al. (2019). They reported a lower total cerebral volume (TCV) in MUO and MUL, but not MHO, as compared to the MHL referent group in a multivariable model adjusted for confounders (age, squared age, sex, and interval between risk factors assessments). This finding suggests that poor metabolic health, rather than obesity status, may be the main determinant for global brain atrophy, in line with other studies (Tiehuis et al., 2014). Furthermore, while (Angoff et al., 2022) reported higher Free water (FW) content and lower FA in several cerebral regions on MHO relative to MHL, suggesting a reduced WM integrity, these differences were more circumscribed than those observed when comparing MUL against MHL. This suggests a more limited impact of obesity status compared to metabolic status, or possibly an earlier stage of brain damage (see also Section “4.1 Methodological limitations in the literature”). While the authors detected no evidence of higher WMH burden or silent brain infarcts in MHO, they observed an impaired global cognitive score, verbal memory, and abstract reasoning in MHO but not in MUL, relative to the referent MHL group. This last finding suggests that obesity, more than unhealthy metabolic profile, was associated with poor cognitive performances even in relatively young adults.

Another direct study investigated the effects of the MHO phenotype on cerebrovascular disease markers in 3,165 neurologically healthy Asian adults (age-range: 50–69 years, 46% women, MHO/MUO/MHL/MUL: 63/1099/588/1415) (Nam et al., 2020). This study examined the prevalence of silent brain infarcts in each group after adjusting for demographic, vascular, and inflammatory confounders -age, sex, current smoking, current alcohol use, anti-platelet agents, high-sensitivity, C-reactive protein (CRP), and white blood cell counts, and found that metabolic status, but not obesity status, was associated with prevalence and burden of silent brain infarcts (i.e., higher burden in MUO/MUL relative to MHL, with no difference between MHO and MHL). It should be noted that this study adopted an Asian-specific definition of MHO, using a BMI cut-off of ≥25 kg/m^2^ to define the obesity status, and a stricter definition of metabolically healthy status that notably included the absence of enlarged waist circumference (WC) with a cut-off of 90 cm in men and 85 cm in women. This definition might thus represent a subclass of the obese population with less risky fat distribution: greater subcutaneous adipose tissue (SAT) than visceral adiposity tissue (VAT) proportions (Nam et al., 2020). Thus, the negative findings on the prevalence of silent brain infarcts could be partially attributed to the healthier profile of MHO in the study.

In elderly samples, results have been more mixed. For example, (Ma et al., 2019) examined T1-w MRI data in a sample of 1,199 non-demented elderly individuals (mean age: 73.5 ± 7.1 years, MHO/MUO/MHL/MUL: 415/363/289/132) from the Alzheimer’s Disease Neuroimaging Initiative (ADNI), a largely North American cohort at the time of the study. Using the NCEP-ATP III definition of MHO, they reported that the MHO subjects (defined using the NCEP-ATP III criterion) showed a decreased AD risk compared with their lean counterpart (MHL), after adjustment for age, sex, genetic (APOE ϵ4) predisposition to dementia, cognitive diagnosis, education, tobacco use, alcohol consumption and LDL-cholesterol (Ma et al., 2019). A further analysis on brain T1-w MRI data reported that whole brain and hippocampal volume were significantly higher in late-life MHO than in MHL groups, thus confirming a protective tendency of the MHO phenotype against the MHL. These findings suggest that, in late life, MHO may paradoxically confer neuroprotection against neurodegenerative processes, even after adjusting for confounders (genetic, clinical, and lifestyle risk factors). The protective tendency of MHO was also corroborated by a higher load of CSF-Amyloid β (Aβ), whose reduction characterizes AD, even after controlling for several possible confounders (Ma et al., 2019).

Of note, existing MHO neuroimaging literature typically does not consider the fact that this status might be a transient phenotype (Ler et al., 2024). To the best of our knowledge, the only longitudinal study on MHO is the one described by Kouvari et al. (2023) only including neuropsychological testing. In Kouvari et al. (2023), 2⋅892 participants from the Framingham Offspring Study (mean age: 60.7 ± 9.4 years, MHO/MUO/MHL/MUL: 234/1152/271/333) were followed longitudinally (12.9 ± 3.5 years) with repeated neurocognitive testing. This study highlighted the transient nature of MHO status, with approximately 70% of participants classified as MHO at baseline developing at least one trait of metabolic disturbance (among dyslipidemia, hypertension and glycemic control) during the follow-up period, and were classified as non-resilient MHO. The remaining participants retained a healthy metabolic status and were thus classified as resilient MHO. The MHO group (defined at baseline) as a whole did not show an accelerated cognitive decline as compared with the metabolically unhealthy groups (MUO/MUL). However, non-resilient MHO showed greater decline in processing speed and executive functions over time compared to resilient MHO participants (Kouvari et al., 2023). Taken together, their findings suggest that obesity or overweight status *per se* are less harmful than the metabolic status for cognitive functioning, in contradiction with evidence reported in Angoff et al. (2022). In other words, the study suggests that long-term maintenance of metabolic health, not obesity status alone, determines resilience against cognitive decline.

The literature on functional neuroimaging in MHO versus MUO is extremely limited. Most fMRI studies to date have focused on obesity as a whole, without stratifying participants by metabolic status. To the best of our knowledge, a single direct study (Dunn et al., 2023) specifically tagged brain activity in MHO, as compared to either MUO or MHL groups. More specifically, (Dunn et al., 2023) carried out a task-fMRI cross-sectional study on 78 participants (mean age: 39 ± 1 years, MHO/MUO/MHL: 29/34/15) stratified using a combination of criteria which included obesity and updated homeostatic model assessment of insulin resistance (HOMA2). They found that insulin resistance and obesity alter dorsal striatal responses (BOLD signals) in an activation task induced by palatable taste. Aberrant neuronal responses to taste in MHO were intermediate between MHL (primarily positive BOLD response) and MUO (primarily negative BOLD response), suggesting that functional brain alterations may parallel the structural and cognitive differences observed between MHO and MUO. Interestingly, lower palatable taste-induced neuronal activation predicted higher food craving and food intake, in line with previous reports of reduced neural response to pleasant food taste in obese versus lean individuals in dorsal striatum (Babbs et al., 2013; Stice et al., 2008). Dorsal striatum integrates cognitive control signals from the prefrontal cortex and homeostatic inputs from hypothalamic regions, and it is thus central for weight control.

No longitudinal fMRI studies have yet determined whether MHO functional brain profiles predict resilience or eventual progression to MUO. Moreover, few studies combine structural and functional modalities, leaving unanswered the question of whether preserved GM/WM integrity in MHO is mirrored by preserved connectivity patterns.

Overall, the few existing direct comparisons of MHO group against MHL do not provide strong evidence for the harmful effects of obesity status in metabolically healthy individuals on MRI-based markers or cognition, with one study reporting protective effects of MHO relative to MHL (Ma et al., 2019), and one reporting harmful effects of MHO relative to MHL (Angoff et al., 2022). Particularly, the lower WM integrity reported by Angoff et al. (2022) in MHO as compared to MHL group was milder in intensity and less widespread than when comparing MUL versus MHL. This suggests the MHO status as a potential intermediate risk phase, as compared to the MUO status. In terms of cognitive functions, while lower global cognitive score in MHO was reported (Angoff et al., 2022), the longitudinal examination of cognitive function in Kouvari et al. (2023) suggests that those who retain a metabolically healthy status have similar cognitive trajectories as MHL subjects, suggesting a limited impact of overweight/obesity status in the absence of other metabolic impairment. However, it should also be noted that only a minority of participants classified as MHO in Kouvari et al. (2023) remained metabolically healthy in the following decade, suggesting MHO as an often-transient status and that the overweight/obesity status as often leading to metabolic disturbances later in life. Their observation is corroborated by several studies following the weight and metabolic status of individuals over time and demonstrating the higher conversion rate of individuals with overweight and/or obesity from metabolically healthy to unhealthy status compared to normal weight individuals (Bogdanov et al., 2020).

#### 3.3.2 Indirect studies

Indirect studies on the MHO are by far more numerous. Among them is a VBM analysis presented by Kharabian Masouleh et al. (2016), which investigated the effects of BMI on GM volume and its cognitive implications on a sample of cognitively healthy subjects with no history of stroke or use of medication affecting the CNS (*N* = 617, age-range: 60–80 years, 42% women, BMI range: 17–41 kg/m^2^) from the Life Adult Study, a predominantly European cohort in Germany (Loeffler et al., 2015). After controlling for prevalent cardio-metabolic conditions, medications, APOE, education status, and WMH traits, their analysis revealed a negative association between BMI and GM volume in multiple cortical areas, including the prefrontal, temporal, and occipital cortices, as well as in subcortical structures such as the thalamus, putamen, amygdala and cerebellum (Kharabian Masouleh et al., 2016). In addition, a mediation analysis revealed that lower GM volume partially explained the association between higher BMI and poorer memory performance and impaired attentional processes (Kharabian Masouleh et al., 2016).

The same group carried out a follow-up analysis (Beyer et al., 2019b) on a partly overlapping sample (*N* = 748, mean age: 68.4 (±4.8), 44% women, BMI range: 17 – 42 kg/m^2^) of cognitively healthy participants with no major brain pathology from the LIFE-Adult Study cohort. VBM-based GM volume, markers of overall (BMI) and visceral (WHR) adiposity, and metabolic features including markers of glucose metabolism (glycated hemoglobin), lipid metabolism (total cholesterol, HDL), systemic inflammation (CRP, interleukin-6), and adipose-tissue derived hormones (leptin and adiponectin) were tested for covariation while controlling for the confounding effects of age, sex, and total intracranial volume (Beyer et al., 2019b). Rather than investigating the independent effects of highly correlated measures of obesity and metabolic status, they applied a multivariate statistical approach to examine the associations between latent factors underlying different measured variables and the GM volumetry. They demonstrated that the higher values of BMI, WHR, leptin, glycated hemoglobin, CRP and lower levels of adiponectin were jointly associated with widespread patterns of decreased GM volume in temporal, frontal and occipital lobe, sub-cortical regions, and cerebellum, suggesting a shared basis among obesity, inflammation, endocrine regulation of appetite and brain alterations (Beyer et al., 2019b). Particularly, BMI, WHR, and systemic inflammation had the highest influence on GM reduction. This study, however, did not control for genetics and behavioral traits, despite a reduced GM volume and impaired executive functions (particularly impulsive behavior leading to disinhibited eating behaviors) might be both secondary to obesity or act as a risk factor for developing obesity later in life (Beyer et al., 2019b; Chuang et al., 2015; Opel et al., 2015).

In the same year, (Lampe et al., 2019) investigated the contribution of obesity to WMH loads using a whole-brain voxel-based approach on a larger sample extracted from the same LIFE-adult cohort (*N* = 1,825, age range: 20–82 years, 44% women, BMI range: 18.4–55.4 kg/m^2^). They found WHR to be robustly associated with deep WMH, after controlling for age, sex, and cardiovascular risk factors including hypertension diagnosis, systolic (SBP) and diastolic (DBP) blood pressure, and smoking. These cardiovascular factors, in turn, were significantly associated with more periventricular WMH. Although BMI was significantly associated with the total WMH volume and the deep to periventricular WMH ratio, the voxel-based analysis did not identify a significant impact of BMI on regional WMH probability, and the effects on the overall WMH volume and regional ratio was uncontrolled for cardiovascular risk factors. The authors in Lampe et al. (2019) also performed mediation analyses and showed that while both BMI and WHR were associated with inflammatory markers like CRP and interleukin-6 (IL-6), only IL-6 significantly mediated the effects of obesity on the WMH ratio. The latter finding aligns with previous studies showing that visceral fat is more linked to inflammation and to microstructural brain damage in deep WM, compared to subcutaneous adiposity (Kim et al., 2017; Widya et al., 2015).

More recently, a comprehensive study conducted on a middle- to older-aged participants of the UK Biobank (*N* = 23,676, mean age: 62.8 ± 7.5 years, 52.5% women, mean WC: 87.9 ± 12.5 cm) examined the impact of obesity in relation to neurodegenerative diseases and metabolic brain aging (Shen et al., 2023). Obesity, defined by elevated waist circumference (WC ≥ 102 cm in males and ≥88 cm in females), showed the strongest association with structural changes in the brain – namely the higher cortical surface area, lower cortical thickness, and lower subcortical volumes-, after adjusting for demographic factors –such as age, sex, ethnicity, and handedness, as well as brain size, socioeconomic and lifestyle factors-. These associations exceeded the influence of other metabolic syndrome components such as hyperglycemia, hypertension, hypertriglyceridemia, high cholesterol and low HDL levels. This result supports the view that obesity defined by WC has an impact on brain aging, beyond other cardiometabolic conditions. The authors also reported significant intercorrelations among all five metabolic components. Importantly, the overall burden of metabolic dysfunction was associated with the magnitude of brain morphological alterations, characterized by reduced basal ganglia volume, increased cortical surface area (CSA), and decreased cortical thickness (CT) in key regions such as the frontal, temporal, and sensorimotor cortices.

Another UK Biobank study (Dekkers et al., 2019) investigated total body fat (TBF), an adiposity measure assessed by impedance, and its effects on brain structural and microstructural integrity (*N* = 12,087, mean age: 62 ± 7.3, 53% women, mean BMI: 26.6 (±4.4) kg/m^2^), stratifying on BMI groups (normal weight, overweight, and obese group). They reported sex-specific effects of TBF, showing a positive association with global cortical volume in women but negative association in men, although there were some specific regional volumes with concordant negative associations in both sexes (in the temporal fusiform anterior cortex and the ventral striatum). Men also exhibited a negative association between TBF and subcortical GM volumes, particularly among the obese group, while in women this association was much weaker and significant only for globus pallidus. In line with this evidence, TBF-by-sex interaction revealed that obese men were more vulnerable to mild cognitive impairment compared to women (Dekkers et al., 2019).

In Caunca et al. (2019a), the link between several indexes of obesity and global MRI-derived markers of brain health was studied in a racially and ethnically diverse North American urban cohort (*N* = 1,289, mean age: 64 ± 8 years, 60% women) including Hispanic/Latino (66%), non-Hispanic Black (17%), non-Hispanic White (15%), and other races/ethnicities (2%). This study revealed significant associations between greater BMI and WC and a reduced CT after adjustment for sociodemographic, lifestyle, cognitive, vascular and metabolic risk factors. Similarly, CT was reduced in obese (BMI > 30) compared to normal-weight participants (BMI < 25), particularly in those younger than 65 years. Concordantly, weaker negative associations were observed for BMI and WC with total cerebral volume, but not with markers of WMH burden. Interestingly, these negative associations between obesity metrics (BMI and WC) and brain atrophy did not change significantly after adjusting for vascular risk factors, suggesting that these factors did not strongly mediate this association (Caunca et al., 2019a).

Taken together, the evidence from indirect studies suggests that both general obesity, as assessed via BMI (Caunca et al., 2019a,b; Kharabian Masouleh et al., 2016), and abdominal (e.g., visceral) adiposity, as assessed via WC or WHR (Caunca et al., 2019a,b; Shen et al., 2023; Suzuki et al., 2019), are negatively associated with markers or regional and global GM volume. Nonetheless, stronger and more robust associations are reported with single or composite measures of visceral adipose tissue [WC, WHR, and multivariate composite measures as in Beyer et al. (2019b)] rather than BMI alone. This may be due to visceral adipose tissue potentially playing a key role in initiating neuroinflammatory pathways that contribute to neuronal loss, as suggested by Lampe et al. (2019) and discussed in Section “3.4 Biological pathways of brain damage.”

### 3.4 Biological pathways of brain damage

**FIGURE 2:**
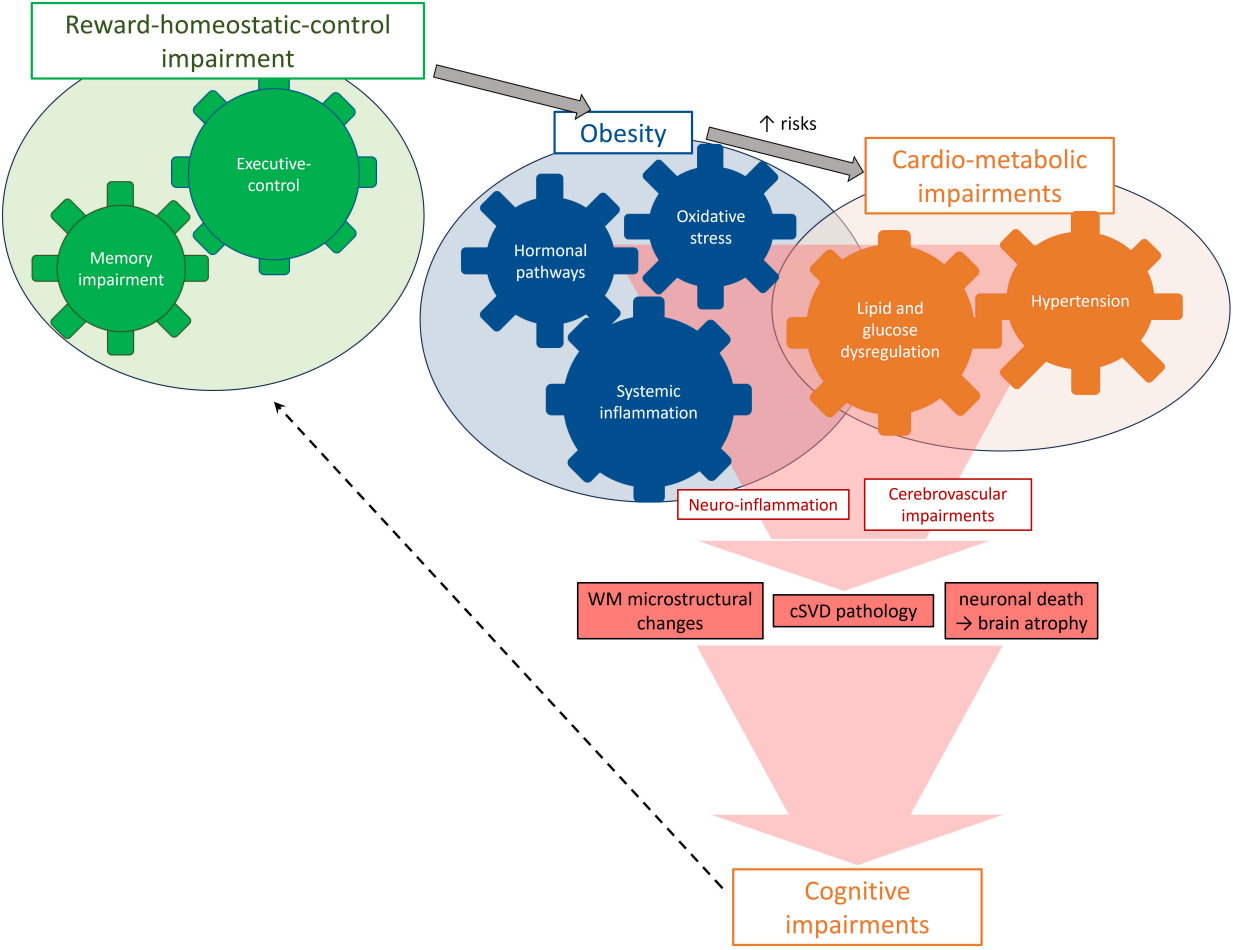
Mechanisms and pathways potentially leading to brain damage in MHO.

Several mechanisms have been suggested as possibly mediating the link between obesity and brain health. Here we summarize key biological pathways that can lead to neuroimaging abnormalities and ultimately to cognitive impairments (Figure 2). Their complex interplay can be modeled using structural equation modeling, as exemplified by Morys et al. (2021), who made use of the large and phenotypically rich UK Biobank (*N* = 20,210, mean age 63 ± 8 years, females: 53%, mean BMI: 26.6 ± 4.27 kg/m^2^) to model the relationships between obesity and other cardio-metabolic measures, brain MRI based measures and cognitive functions. This study revealed that (i) obesity was related to systemic inflammation and metabolic comorbidities of obesity like hypertension, diabetes, and dyslipidemia; (ii) these, in turn, were associated with cerebrovascular alterations (WMH), which were further related to cortical thinning, GM loss and impaired cognition; and (iii) obesity measures (BMI, WHR and BF%) were also directly related to GM shrinkage independent of WMH. Below we review obesity-related pathways (Sections “3.4.1 Inflammatory pathways: systemic low-grade and neuro-inflammation,” “3.4.2 Hormonal pathways: leptin, adiponectin and ghrelin,” “3.4.3 Oxidative stress”), then cardio-metabolic factors likely triggered by obesity (Section “3.4.4 Cardio-metabolic pathways: blood pressure, lipid, and glucose homeostasis”), and their collective impact on the cerebrovascular health (Section “3.4.5 Cerebrovascular pathways”).

#### 3.4.1 Inflammatory pathways: systemic low-grade and neuro-inflammation

Adipose tissue in the abdominal region can be considered as an active endocrine organ secreting pro-inflammatory cytokines, such as interleukin-6 (IL-6), and inflammation-related proteins, such as CRP (Morys et al., 2021). These cytokines promote a status of chronic low-grade inflammation, endothelial dysfunction, and disrupted fibrinolysis (Hakim, 2019; Kivimäki et al., 2022). These have been shown to increase the permeability of the blood-brain-barrier to circulating cytokines and immune cells, paving the way for neuroinflammation (Chiefari et al., 2021; Yaffe et al., 2004). In turn, neuroinflammation can impair cognition, learning-related skills, and social behavior, potentially leading to neuropsychiatric disorders (Biessels and Despa, 2018). Interestingly, elevated markers of systemic inflammation (such as IL-6 and CRP) have been linked to increased dementia risk (Yaffe, 2007; Yaffe et al., 2004), even decades prior to its occurrence (Schmidt et al., 2002), possibly due to accelerated accumulation of amyloid-β (Abdallah et al., 2021; Keaney et al., 2015; Ma et al., 2018). In accordance with this, markers of systemic inflammation have been also linked to MRI-derived markers of global brain atrophy in middle-aged adults (Gustafson et al., 2003), to GM reduction particularly in the hypothalamus, hippocampus and prefrontal cortex (Marsland et al., 2008), and to macroscopic WM markers of cerebral vessel disease (Hakim, 2019). This is in line with, previous animal studies showing that highly fatty diets induce blood-brain-barrier breakdown, hypothalamic inflammation and subsequent atrophy, whereas calorie restriction reduces systemic inflammation and brain atrophy (Hargrave et al., 2016; Kanoski et al., 2010; Kanoski and Davidson, 2011; Thaler et al., 2012; Willette et al., 2010).

#### 3.4.2 Hormonal pathways: leptin, adiponectin and ghrelin

Leptin, adiponectin and ghrelin are signaling hormones playing a key role in energy homeostasis that also influence brain structure and function (Angoff et al., 2022; Beyer et al., 2019a; Han et al., 2021). In the hypothalamus, a group of nuclei respond to these hormonal signals to either promote or suppress feeding (arcuate nucleus), to regulate energy expenditure (paraventricular nucleus), to activate food-seeking and rewards-related behaviors (lateral hypothalamus), and to activate satiety signals (ventromedial hypothalamus) (Purnell and le Roux, 2024). Dysregulation of these nuclei has been consistently observed in both rodent-models of diet-induced obesity and in humans, and linked to abnormal feeding behaviors and energy imbalance (Briggs et al., 2010; Enriori et al., 2007).

Besides being involved in the hypothalamic control of satiety signals and suppression of food intake, leptin also acts in the hippocampus to support memory functions (Beyer et al., 2017; Klok et al., 2007). Increased levels of leptin have been associated with reduced dementia incidence in non-obese adults and larger brain volume (Lieb, 2009). On the other hand, chronically elevated levels of leptin in obesity induce resistance to the effects of the molecule (Myers et al., 2010) and have been linked to impaired executive functions in older adults (Angoff et al., 2022; Spitznagel et al., 2010). In line with this, because of the higher expression of leptin in subcutaneous fat tissues, this hormone has been hypothesized to be a less important neurodegenerative risk factor compared to visceral adiposity (Debette et al., 2010).

Adiponectin has positive effects on the brain due to its insulin sensitizing (Cisternas et al., 2019) and glucose-regulating properties (Lihn et al., 2005). However, existing neuroimaging studies have failed to find evidence of a link between adiponectin and GM volume (Beyer et al., 2019b; García-Casares et al., 2016).

Ghrelin stimulates meal-initiation and thus food intake, but also affects brain regions controlling rewards and mood regulation such as prefrontal cortex and striatum (Bogdanov et al., 2020). Beyond feeding, ghrelin has also been found to have a neuroprotective effect after ischemic brain injury (Spencer et al., 2013) and brain traumatic injury in mice (Shao et al., 2018), possibly due to prevention of blood–brain barrier breakdown and neuronal death. Ghrelin is inversely correlated with BMI (Klok et al., 2007), and its metabolic action appears dysregulated in obesity, possibly due to ghrelin resistance.

Finally, high levels of cortisol, a steroid hormone usually secreted under stress conditions and oversecreted in subjects with obesity (Björntorp, 2001) might lead to premature brain atrophy (Simmons et al., 2000).

#### 3.4.3 Oxidative stress

Obesity increases oxidative stress - an imbalance between generation and clearance of reactive oxygen species (ROS) and reactive nitrogen species (RNS) – through biochemical mechanisms such as superoxide generation and oxidative glyceraldehyde auto-oxidation (Čolak and Pap, 2021; Wang, 2010). These in turn trigger further deposition of adipose tissue by promoting preadipocyte proliferation, adipocyte differentiation and growth (Čolak and Pap, 2021; Dandona et al., 2010; Higuchi et al., 2013). Oxidative stress also impacts insulin secretion and glucose transport in adipose tissue and muscles, being therefore involved in the development of metabolic disturbances (Čolak and Pap, 2021; Hopps et al., 2010), and has also been linked with increased permeability of the blood-brain-barrier (Čolak and Pap, 2021).

Due to its high metabolic activity, the brain is particularly vulnerable to oxidative damage (Reynolds et al., 2007; Singh et al., 2019). Cerebral oxidative stress causes insults to neurons, such as hypoxia and hypoglycemia, leading to cell injury and neuronal dysfunction in specific population of neurons more vulnerable to age-associated decline -particularly in the hippocampus, amygdala, and prefrontal cortex-, with consequent behavioral and cognitive impairment (Salim, 2017; Wang, 2010). This ultimately leads to neuropsychiatric diseases such as depression (Correia et al., 2023), and to neurodegenerative disorders such as Alzheimer’s, Parkinson’s, and Huntington diseases, as well as amyotrophic lateral sclerosis (Reynolds et al., 2007; Singh et al., 2019; Wang, 2010).

#### 3.4.4 Cardio-metabolic pathways: blood pressure, lipid, and glucose homeostasis

Central obesity – prominently hypertrophic adipocytes - has been linked to the disruption of insulin signaling and insulin resistance, possibly via diet-induced inflammation (Medic et al., 2016; Vachharajani and Granger, 2009; Velloso, 2009). Notably, neuronal cells influenced by insulin can affect critical CNS functions, including neurotransmission, synaptic plasticity, and neuroprotection (Fanelli et al., 2022; Klinedinst et al., 2019). Disturbed insulin signaling pathway and consequent glucose dysregulation have been also linked to impaired memory and to GMV reduction in key memory regions such as the hippocampus and the temporal lobe (Beyer et al., 2019a; Cherbuin et al., 2012; Kerti et al., 2013). Concordantly, disturbed insulin signaling has been thought to be implicated in the pathogenesis of neurodegeneration (Wrighten et al., 2009), particularly of AD (Kroner, 2009).

Overall, this evidence suggests a pathway linking obesity and cognitive impairments, through cardio-metabolic dysfunction and neuroanatomical alterations.

#### 3.4.5 Cerebrovascular pathways

Obesity has also been linked to maladaptive changes of the vasculature (O’Rourke and Safar, 2005), including arterial stiffness, thickening of the carotid wall, ventricular hypertrophy, vascular endothelial dysfunction, hypertension, and ischemia (Taki et al., 2008). These alterations can compromise cerebral perfusion and damage brain tissues. In particular, cerebral vascular pathology has been associated with decline in GM tissues, especially in the hippocampus and lateral temporal lobe (de Toledo Ferraz Alves et al., 2010; Tini et al., 2020). Each standard deviation increase in carotid-femoral pulse wave velocity corresponds to a reduction in total cerebral volume (TCV) equivalent to ∼1.2 years of accelerated brain aging (Tsao et al., 2013).

In Morys et al. (2021), cerebrovascular alterations were also associated with cognitive impairments and the link between BMI and working memory was partly mediated by WMH load (9%), as were the associations of WHR with working memory (7%) and fluid intelligence (21%), and the association between BF% and working memory (9%). In WM, vascular dysfunction might lead to demyelination, loss of oligodendrocytes, and gliosis (Morys et al., 2021), which might accumulate in the years and result into increased WMH load and/or into a sensible change in other markers of WM pathology, such as silent cerebral infarcts (de Toledo Ferraz Alves et al., 2010; Tini et al., 2020; Tsao et al., 2013).

Vascular stiffening might also lead to hypoperfusion and hypoxia, and cause cerebral tissue damage (Lampe et al., 2019). A significant clinical consequence of cerebrovascular pathology is the onset of cognitive impairment and increased risk of dementia, even in otherwise healthy individuals (Friedman et al., 2014; Furlano et al., 2021; Wrighten et al., 2009). A recent study in the field (Siedlinski et al., 2023), combining genetic and observational evidence, led to similar conclusions, supporting a negative influence of elevated blood pressure on cognitive performance also through genetic causal inference methods (Mendelian randomization). Notably, the latter study also identified deleterious effects of systolic blood pressure on t neuroimaging markers of cognitive function, especially in the anterior thalamic radiation, the anterior corona radiata, and the external capsule (Siedlinski et al., 2023).

## 4 Discussions and conclusions

There is no clear characterization of the neuroanatomical and neurofunctional signature of the “metabolically healthy obese brain” and of its neurocognitive characteristics. Comparative studies between metabolically healthy (MHO) and unhealthy obese (MUO) reveal lower GM (Angoff et al., 2022) and WM (Nam et al., 2020) integrity and worse cognitive outcomes (Kouvari et al., 2023) in the latter group, highlighting the critical role of metabolic status in determining brain health in individuals with obesity. Nonetheless, there are also indications that MHO individuals are still at increased risk of impaired brain health when compared to their lean counterparts (MHL), as reported in three out of the four direct studies reviewed (Angoff et al., 2022; Kouvari et al., 2023; Nam et al., 2020) This points to a potential role of obesity, and overall fat composition more in particular, in disrupting brain health even in the absence of overt cardio-metabolic disturbances. It also supports the notion that preserving metabolic health in individuals with obesity has a measurable protective effect on brain health. This perspective underscores the potential of neuroimaging techniques to unveil distinctive patterns of subclinical neuroanatomical changes associated with diverse health conditions accompanying obesity, offering valuable insights into the intricate relationship between psychiatric, neurological and systemic disorders. Given the transient nature of the MHO phenotype into MUO for most (but not all) individuals, the MHO could be seen as a precious time window for interventions aimed at preserving a healthy metabolic status and preventing the obesity-related brain damage (Blüher, 2020, 2010; Roberson et al., 2014).

### 4.1 Methodological limitations in the literature

Despite the evidence reported, the literature in the field is fairly inconsistent, possibly due to different sources of heterogeneity across studies, which might explain, at least in part, the obesity paradox. We provide below an overview of such aspects.

#### 4.1.1 Heterogeneity in cognitive assessment methods

Among the instruments summarized in Table 5, the Montreal Cognitive Assessment (MoCA) stands out for its greater sensitivity compared with the Mini-Mental State Examination (MMSE) in detecting subtle deficits, particularly in fronto-executive domains (Freitas et al., 2013; Nasreddine et al., 2005). Because obesity and metabolic dysregulation are thought to preferentially affect prefrontal circuits involved in executive control (Rösch et al., 2020), the use of the MoCA or other sensitive instruments is essential to capture early brain–behavior changes.

The lack of standardized cognitive batteries, combined with the frequent use of screening tools with different cut-offs and follow-up periods, further complicates comparisons across studies and may partly explain the variability of neurocognitive findings. Therefore, when interpreting cognitive outcomes in this literature, it is important to consider both the psychometric properties of the instruments and the specific domains they assess, as these factors strongly influence the detection of subtle deficits and the characterization of longitudinal cognitive trajectories in individuals with obesity.

#### 4.1.2 Obesity definition via BMI

Despite its wide use, the definition of obesity via BMI has some obvious limitations since it cannot discriminate lean from fat mass (body composition), nor subcutaneous adiposity from either visceral or ectopic fat deposition (body-fat distribution) (De Lorenzo et al., 2019; Hainer and Aldhoon-Hainerová, 2013; Kassir et al., 2023; Macek et al., 2020; Smith et al., 2019; Tchernof and Després, 2013). This is especially important when studying the elderly (Huxley et al., 2008; Kim et al., 2017), since visceral adipose tissue deposition tends to increase significantly with age (Smith et al., 2011; Tchernof and Després, 2013) while skeletal muscle mass is progressively reduced (i.e., sarcopenia) (Guglielmi et al., 2016). Moreover, there is a difference between males versus females, due to body composition, when computing BMI (Pray and Riskin, 2023). Consequently, other obesity indexes like the accumulation of visceral adipose tissue (VAT) measured from abdominal CT images and the % BF computed from Dual-Energy X-ray Absorptiometry, have been proved to be better predictors of clinical and neurocognitive health outcomes (Kim et al., 2017), especially in the case of late-onset obesity (Hainer and Aldhoon-Hainerová, 2013).

Previous reviews have highlighted that fat distribution cannot be overlooked, as excess visceral and ectopic adiposity (central obesity), more than subcutaneous fat accumulation (peripheral obesity), are linked to dyslipidemia, proinflammatory and prothrombotic activity, increased risk for cerebrovascular disease, more severe microstructural brain damage and more adverse cognitive outcomes (Hainer and Aldhoon-Hainerová, 2013; Iacobini et al., 2019; Kim et al., 2017; Lampe et al., 2019; Tchernof and Després, 2013; Widya et al., 2015). Interestingly, (Debette et al., 2010) reported a negative association between VAT and total brain volume, independent of BMI in middle-aged community participants.

#### 4.1.3 MHO definition

The prevalence of MHO has been reported to range from approximately 6% to 75% of the total adult obese population (Eckel et al., 2016; Ma et al., 2019; Rey-López et al., 2014; Smith et al., 2011), depending on the employed criteria applied. In other words, the definition of the MHO phenotype is not unique, mostly due to differences in the number and severity of metabolic abnormalities included across the different definitions. Therefore, this represents an aspect which might have contributed to the high heterogeneity of findings reported in the obesity literature (Eckel et al., 2016; Gómez-Zorita et al., 2021; Ma et al., 2019; Rey-López et al., 2014; Smith et al., 2011).

Furthermore, the most commonly used definitions of MHO phenotype suffer from the inherent limitations of dichotomous classifications: (1) a small variation in one parameter can lead to classify a subject into an opposite class/category; (2) subjects within the “metabolically unhealthy” class are considered as equally affected (same level of “sickness”), without taking into account the number or severity of metabolic traits; (3) subjects within the “obese” class are considered equivalent in terms of adiposity (same level of “fatness”), without taking into account its severity and distribution; (4) subjects which do not meet yet a clinical diagnosis for any metabolic trait but express sub-clinical disturbances are all classified as “metabolically healthy.”

Finally, the two most common MHO definitions in the neuroimaging literature (namely the NCEP-ATP III and the IDF criteria) do not incorporate markers of systemic inflammation and/or hormonal imbalance, neglecting the importance of these molecular mechanisms as possible pathways underlying brain damage in metabolically healthy and unhealthy obesity.

#### 4.1.4 Sex effects

Adipose tissue tends to distribute differently between sexes, with visceral fat more common in men and subcutaneous fat more common in women (Nakamura et al., 1994; Taki et al., 2008). Furthermore, women tend to be more insulin-sensitive than men, possibly due to their higher levels of adiponectin, and/or to the effect of estrogens onto insulin and glucose homeostasis (Cavalieri et al., 2010; Geer and Shen, 2009; Kim, 2024). Although prevalence estimates vary depending on the diagnostic criteria employed, the MHO phenotype is more common in women than in men (Smith et al., 2019). As a further confirmation, previous neuroimaging studies have suggested that the relationship between GM atrophy and obesity might be modulated by gender (Kurth et al., 2013; Taki et al., 2008). This calls for a more careful treatment of sex, which may likely act as an effect modifier.

#### 4.1.5 Differences in sensitivity of MRI-derived biomarkers

Different MRI markers are sensitive to different anatomical/functional changes which might happen at different times and at a different spatial (microscopic versus macroscopic) scale. As an example, DTI-derived markers of WM integrity can detect earlier and more spatially subtle alterations than cSVD markers from T2w-MRI, which typically indicate macroscopic WM anomalies. More so, cSVD induces protracted stenosis of small arteries, that leads to hypoperfusion of the brain, resulting first in small cerebral infarcts and WMH loads, and only later into cerebral atrophy due to the accumulation in time of brain damage (Hakim, 2019). It follows that inconsistent neuroimaging findings might partially be due to the timing that subjects have been imaged during their lifespan, and to the MRI-modalities used to examine anomalies.

#### 4.1.6 Causality and bidirectionality: the need for longitudinal studies

Most of the reviewed studies are cross-sectional (Table 6). This approach underlines the assumption that MHO is a stable rather than transient state, despite previous epidemiological evidence indicating the transitions for many individuals either in metabolic health or obesity status over time (Bobbioni-Harsch et al., 2012; Eckel et al., 2016; Janssen et al., 2004; Ler et al., 2024; Soriguer et al., 2013). Also, subjects with a later onset of metabolic dysfunction and shorter exposition to the obesity insult might be representative of an earlier stage of the trajectory, and this in turn might explain different neuroimaging (and neurocognitive) findings (Hainer and Aldhoon-Hainerová, 2013). Furthermore, cross-sectional studies implicitly assume that the observed neuroanatomical, neurofunctional, or cognitive alterations are consequences of the obesity insult. However, emerging evidence suggests that abnormalities in rewards- and control-related regions (e.g., such as atrophy and/or altered activation in the prefrontal, orbitofrontal cortex, striatum, and anterior cingulate) might actually precede obesity. These abnormalities might then predispose subjects to overeating behavior and impaired self-regulation, and thus to weight gain (Lowe et al., 2019; Stice et al., 2008; Yokum et al., 2011). This could further exacerbate brain damages in these same regions (Lowe et al., 2019; Stice et al., 2008; Yokum et al., 2011).

In this view, longitudinal studies may help to untangle the temporal direction of effects and clarify if executive dysfunction might be a cause of obesity, a consequence, or both. Statistic approaches that take advantage of genetic instruments, such as Mendelian randomization, may allow us to clarify the causal relationship between obesity sub-types and brain trait variability, in a way free of typical biases of observational studies like residual confounding and reverse causality (e.g., increased mortality among subjects with more risky fat distributions, and weight loss associated with a preclinical dementia status - see Section “4.3.2 Recommendations for future research: toward a precision obesity approach”).

#### 4.1.7 Population age and other sources of bias

It has been postulated that subjects with a less favorable obesity, both in terms of degree and fat distribution, tend to have a shorter life expectancy than obese subjects with less risky (subcutaneous lower body) obesity patterns (Beyer et al., 2017, 2019a). Furthermore, unintentional weight loss has been reported to precede AD diagnosis in older adults (Johnson et al., 2006), and to be exacerbated as dementia progresses (Franx et al., 2017). Age and other differences in the sample demographics, as well as limited sample sizes, might therefore partly account for the discrepancies in the literature (Kharabian Masouleh et al., 2016). In addition, since obesity is associated with increased head motion during scanning and thus image artifacts, special attention should be paid to control for head micro-movements during image acquisition (Medawar and Witte, 2022). Education, smoking habit, and cardiorespiratory fitness status are further factors that might confound the study of possible links among adiposity metrics, MRI markers and cognitive performances, as are the possible concurrent presence of other clinical/subclinical conditions (e.g., depression) and related medication intake, which are not accounted for by the most used MHO definitions (Kharabian Masouleh et al., 2016). Finally, collider stratification bias, survivorship bias and other potential methodological aspects may explain the obesity paradox and definitely deserve careful consideration (Banack and Kaufman, 2013).

#### 4.1.8 Generalizability to non-Caucasian ethnicities and health equity concerns

Most neuroimaging studies on obesity and brain health have been conducted on Caucasian cohorts from high-income countries, mainly in North America and Europe. Compared to Caucasian and European populations, Asian populations typically exhibit higher central obesity for each stratum of BMI (Huxley et al., 2008), which may lead to different susceptibility to metabolic complications and brain tissue damage (Kim et al., 2017). Similar ethnic differences in body composition, fat distribution, and metabolic profiles exist in African, Hispanic/Latino, and Indigenous populations (Martos-Moreno et al., 2020; Justice et al., 2021). Besides minority ethnic groups, individuals from lower socioeconomic backgrounds face higher risks of obesity, metabolic disturbances, and cognitive aging possibly due to dietary, cultural, and/or environmental factors (García-García et al., 2022; Tchernof and Després, 2013), yet they are typically under-represented in neuroimaging studies. This ethnic, geographic, and socioeconomic imbalance limits the generalizability of findings to these populations. Beyond methodological limitations, this lack of diversity also raises ethical concerns related to health equity.

#### 4.1.9 Rebound mechanisms in obesity and brain health

Weight-loss interventions, whether behavioral, pharmacological, or surgical, are often followed by compensatory “rebound” responses that tend to restore body weight (Contreras et al., 2019; Strohacker et al., 2014). These include reduced resting energy expenditure, increased appetite mediated by ghrelin, and decreased satiety hormones such as leptin and GLP-1, as well as heightened activity of hypothalamic–pituitary–adrenal stress pathways (Aschbacher et al., 2014; van Loenen et al., 2022). Such homeostatic adaptations favor rapid weight regain and recurrent metabolic stress, which in turn may exacerbate systemic inflammation, insulin resistance, and cerebrovascular dysfunction (Phuong-Nguyen et al., 2024). Repeated weight cycling (“yo-yo” dieting) has been associated with greater visceral fat accumulation and impaired WM integrity, potentially accelerating age-related brain atrophy and cognitive decline. Considering rebound physiology is therefore critical when interpreting neuroimaging findings in obese individuals, particularly in those undergoing weight-loss interventions. For these reasons, our review does not also cover samples of individuals who have undergone significant weight-lost interventions.

### 4.2 Strengths and limitations

Despite this review presents a clear aspect of novelty by attempting to compare direct versus indirect studies investigating the impact of obesity onto brain health, several limitations should be acknowledged. First, we were unable to make strong and definitive conclusions on the possible associations between obesity, metabolism status, and brain health, a limitation largely due to current limitations of the research field (see Section “4.1 Methodological limitations in the literature” above). Indeed, the scarcity of available studies, the inconsistencies across reported findings, the heterogeneity across the MHO definitions, the relatively small and demographically narrow sample sizes, and the predominantly cross-sectional nature of most of the current scientific literature, necessarily limited the strength of our conclusions. Second, information on silent brain disease, such as silent strokes or subclinical cerebrovascular events, is typically not provided and we cannot exclude confounding effects. Finally, as this is a narrative review, publication bias cannot be excluded.

### 4.3 Future directions and clinical implications

#### 4.3.1 Eating behavior and its neuronal control

While it is acknowledged that obesity, with or without other cardio-metabolic comorbidities, might cause measurable neuroanatomical and neurocognitive damage, the possible influence of neuroanatomical and neurocognitive alterations on incident obesity remains an open and intriguing question. Indeed, it has been hypothesized that anatomical changes in the prefrontal cortex, a brain region supporting executive functions and under genetic control (Chouinard-Decorte et al., 2014), might precede the development of overeating - through deficits of self-regulatory control over food intake - and thus obesity (García-García et al., 2019). These same neuroanatomical alterations might then be further exacerbated by the obesity insult in a vicious cycle of progressive obesity severity, brain dam-age and cognitive decline (Hargrave et al., 2016). Similar positive feedback has been hypothesized for the hippocampus (Hakim, 2019), possibly initiated by hippocampal dysfunctions due to congenital deficits, early exposition to highly-caloric fatty diets, and/or to environmental toxin exposition (Hargrave et al., 2016; Parent et al., 2014; Shefer et al., 2013). In turn, hippocampal dysfunctions might cause impaired memory inhibition leading to unhealthy responses to food cues, consumption of energy dense food, and in turn weight gain (Dye et al., 2017).

Most of the current knowledge of neuronal control on eating behaviors is coming from animal studies. In humans, task-based functional MRI studies have reported differences in the activation patterns of individuals with obesity when presented with food stimuli, that possibly would make them more inclined to high-caloric food craving and thus to obesity (Rothemund et al., 2007). Aberrant brain networks and related cognitive functions - such as rewards evaluation and eating behaviors - are also important aspects in obesity research. Indeed, network connectivity studies from resting-state MRI have found that BMI is negatively associated to posterior default mode functional connectivity in older adults - independently of possible cardio-metabolic comorbidities, gray matter volume and APOE genotype - and with worse executive functions and outcomes (Beyer et al., 2017). Interestingly, decreased posterior default mode connectivity has been also reported in cognitively normal adults at increased genetic AD risk (APOE-e4 carriers) (Sheline et al., 2010), and in subjects presenting mild cognitive impairment (Sorg et al., 2007), suggesting that obesity might be associated to patterns of functional connectivity abnormality similar to those observed in individuals at AD risk (Beyer et al., 2017; Sheline et al., 2010).

#### 4.3.2 Recommendations for future research: toward a precision obesity approach

To avoid the aforementioned shortcomings of current neuroimaging literature on the MHO phenotype (see Sections “4.1 Methodological limitations in the literature” and “4.2 Strengths and limitations”), we recommend that future studies should be carried out in large multicenter cohorts and in a longitudinal setting, following in time both MHO subjects who will convert into metabolic unhealth (MHO converters) and those who will not (MHO resilients) (García-García et al., 2019). The use of multi-modal (including both structural and functional) and multi-scale (both microscopic and macroscopic) brain imaging, might allow a more comprehensive understanding of the shared underpinnings of obesity, metabolic regulation, and brain health (Petersen et al., 2024). In particular, more functional MR imaging studies (such as rs-fMRI and task-MRI), which remain underutilized in this field, might fill critical gaps in understanding network-level brain changes in obesity. Also, systematic meta-analyses might provide complementary information on the effect sizes and direction of effects across different cohorts and imaging modalities. Furthermore, we recommend the use of a standardized criterion to define the MHO phenotype that would capture the truly multifaceted nature of obesity, especially of visceral and ectopic adiposity, and related metabolic comorbidities, in each of its facets/dimensions: severity and distribution of fatness, vascular health, glycemic control, dyslipidemia, low-grade systemic inflammation, hormonal dysregulation and resistance, ethnicity, cardio-metabolic fitness, lifestyles. Given the limitations of BMI as a proxy for adiposity (see Section “2 Metabolically healthy obesity (MHO)”), we also recommend that future neuroimaging research on obesity adopt more biologically informative measures of obesity, such as waist circumference, waist-to-hip ratio, or imaging-based assessments of visceral and subcutaneous fat distribution. Stratifying future analyses by sex/gender or other potential modifiers (e.g., polygenic influences on obesity, neuroimaging traits, eating behaviors, and socioeconomic status) might also improve the sensitivity of future studies. Similarly, integrating genetic information through the use of polygenic scores or, even better, instrumental variables in two-sample Mendelian randomization studies, might help uncover possible causal links in this complex relationship.

Studying obesity status through these lenses and its interplay with brain imaging endophenotypes may contribute to furthering our understanding of the pathological processes associated with neurodegenerative and neuropsychiatric diseases. Most importantly, it may represent one step forward in establishing features for the early diagnosis of neurodegenerative conditions, which currently suffer from the lack of risk-predictive biomarkers. If MHO converters, a subset of the adult MHO population at high risk of developing brain health complications, could be identified early enough, this information could be used in clinical settings. Also, targeted public health strategies could be planned for weight loss in these subjects, before brain (and cardio-metabolic) health is irreversibly impaired. The use of risk-stratified treatment for obesity may possibly lead to a consequent reduction of the prevalence and burden of both obesity and of its neurological and neuropsychiatric sequelae, which have currently reached epidemic diffusion in most populations, especially among elderlies, with unprecedented costs for most welfare and healthcare systems.

### 4.4 Conclusion

In this narrative review, we integrated evidence of neuroanatomical abnormalities– as derived from structural brain MRI data– with findings of disrupted brain networks and activation patterns –from functional brain MRI data– in metabolically healthy obesity, examining their links to cognitive dysfunctions. By doing so, we comprehensively covered profound aspects of the complex relationship among obesity, metabolic health, and brain health, and emphasized their relevance for both clinical and research purposes. Furthermore, by jointly reviewing evidence from both structural and functional imaging data, we aimed to move beyond a single modality approach, offering a broader view of how obesity may shape brain health.

However, the evidence collected here is partly contrasting, with the majority of studies directly comparing MHO and MUO supporting a neuroprotective or cognitively advantageous profile for MHO individuals and other studies reporting either no difference or mixed findings. Differences in sample demographics and the use of an oversimplified definition of metabolically healthy obesity (which overlooks the type and degree of obesity, as well as a possible inflammatory status, hormonal control, lifestyle factors, genetic risk for dementia, medication intake, and subclinical metabolic disturbances), are among the most important sources of bias. Findings reported here partially support the view of adipose tissue as an active endocrine organ damaging the CNS - independently of the comorbidities which might coexist with obesity - even if in a milder form compared to metabolically unhealthy obesity. They also highlight the key role of alterations in rewards-homeostatic-control networks. While firm conclusions on the MHO phenotype and its effects on brain health cannot yet be derived, the evidence collected here warrants further systematic investigations on large sample sizes, via multimodality structural and functional neuroimaging, the use of a more comprehensive and standardized definition for MHO, a careful assessment of cognitive dysfunction, and the investigation into possible causal pathways linking obesity, cardio-metabolic disturbances, and brain structural/functional damage.

To conclude, in a public health context of global aging populations and rising prevalence of obesity, the present review underscores the urgency of better understanding the effects of obesity on brain health for implementing prevention strategies targeted at promoting healthy cognitive aging.
