# Supplementary material for: How is the brain affected by metabolically healthy or unhealthy obesity in adulthood and elderly? A narrative review of neuroimaging and neurocognitive findings
Source: Front Aging Neurosci. 2025 Dec 1;17:1616303. doi: 10.3389/fnagi.2025.1616303 (PMC12703103; doi:10.3389/fnagi.2025.1616303)
Supplement: Supplementary file 1 [file Table_1.docx]

Supplementary Material

# Voxel-Based Morphometry (VBM)

## Image processing pipeline

Voxel-based morphometry (VBM) approach is an established automatic quantitative technique used for voxel-wise investigation of brain tissues composition (volume or density) throughout the entire brain. The imaging processing pipeline of VBM, as implemented in SPM software package (SPM, version 12, Wellcome Department of Cognitive Neurology, London, United Kingdom, https://www.fil.ion.ucl.ac.uk/spm/), has been extensively described elsewhere (Ashburner and Friston, 2000; Wright et al., 1995) and is shortly summarized here.

At first, longitudinal (intra-subject) or population (across-subjects) MR brain images are spatially normalized into a common reference space with linear and then non-linear warping of the images, and corrected for intensity non-uniformities. Next, a priori probability maps are used for probabilistic segmentation of brain voxels into its three main tissue types: GM, WM, and CSF. Tissue maps can be modulated by the Jacobian determinants of the former non-linear normalization to account for possible stretching and compression of the images. Next, brain tissue’s volume (modulated maps) or density (non-modulated maps) maps are smoothed by convolution with a half maximum isotropic Gaussian kernel. This step improves normality, and accounts for both inter-subject variability in gyral anatomy and registration errors. Finally, whole-brain tissue maps can be analyzed via regression models with mass univariate statistics at each voxel, or averaged VBM values can be computed for regions of interest.

## Metrics

VBM has been used to detect subtle GM changes associated with aging and neuropathology via mass univariate statistic of voxel-level metrics (Good et al., 2002). Otherwise, summary statistics of brain composition can be derived for specific regions of interest (ROIs) with a known functional specialization (Roy et al., 2020), as well as at hemispheric or global level to derive phenotypes such as total GM volume and the total WM volume. Partial least squares correlation (PLSC), a multivariate statistical method that can handle highly spatially correlated data (Grellmann et al., 2015), can also be used to derive patterns of covariation between VBM’s voxel-level tissue maps and other variables of interest.

Notice how the use of VBM approach for studying cortical volume has some important limitations due to sub-optimal alignment that does not take into account the individual sulci/gyri positions (Lyttelton et al., 2007; Maingault et al., 2016).

# Surface Based Morphometry (SBM)

## Image processing pipeline

The imaging processing pipeline of SBM as implemented in FreeSurfer (https://surfer.nmr.mgh.harvard.edu/fswiki/FreeSurfer) has been extensively described elsewhere (Fischl and Dale, 2000; Good et al., 2002; Rakic, 1995a) and is shortly summarized here.

The first image processing steps involve intensity non-uniformities removal, skull stripping, and segmentation of GM, WM, and CSF tissue types. After the two cerebral hemispheres are separated, inner (at the intersection between of GM and WM) and outer cortical surfaces are extracted and corrected for topological defects. Next, brain morphometric traits are estimated in the native anatomical space of the subject. Once an accurate matching of homologous locations is established across cortical surfaces via high-dimensional warping, these morphometric indexes can be studied locally (vertex-level), or averaged either in regions of interests —most typically the Desikan-Killiany-Tourville (DKT) atlas (Klein and Tourville, 2012) — or at hemispheric and/or global level (Medic et al., 2016).

## Metrics

At the heart of SBM is the computation of a number of vertex-level morphometric metrics, including cortical thickness (CT) and cortical surface area (CSA), cortical volume (CV), cortical curvature, gyrification and sulcal depth in the two hemispheres. In FreeSurfer, CT is computed as the shortest distance between the inner and outer cortical surface models for each corresponding vertex, while CSA is computed at each vertex of the inner (or outer) surface model as the average area of the triangles to which that vertex belongs. Of particular interest are CT and CSA, since they have partly different genetic determinants (Panizzon et al., 2009; Rakic, 1995b), exhibit different trajectories during the lifespan, and their abnormalities are associated to different cognitive impairments and disorders (Lee et al., 2016; Schnack et al., 2015; Vuoksimaa et al., 2016)— for reviews see (Meyer et al., 2014; Winkler et al., 2018). On the other hand, CV has important limitations due to the fact that it is computed as the product of CT by CSA, which are independent anatomical traits commonly anti-correlated to each other (Maingault et al., 2016; Meyer et al., 2014; Winkler et al., 2018; Worker et al., 2014).

# DTI processing

## Image processing pipeline

DTI data is most typically processing is typically performed via well-established methods such as the ones described in (Jenkinson et al., 2012).

## Metrics

Fractional anisotropy (FA), mean diffusivity (MD), axial diffusivity (AxD), and radial diffusivity (RD) are some of the most used DTI-derived metrics that can quantify microscopic WM integrity by studying the dynamics of water molecules diffusion in WM tracts. Low values of FA and high values of MD are suggestive of decreased levels of WM integrity, possibly reflecting demyelination, damage to the axon membrane, and/or reduced axonal packing density/coherence (KUBICKI et al., 2007; Sanjari Moghaddam et al., 2019; Shenton et al., 2012). Evidence of impaired WM integrity is a phenomenon often encountered in the elderly, as well as in patients with cardiovascular disease and type II diabetes (Lamar et al., 2020), (Den Ruijter et al., 2012; Han et al., 2021; Ma et al., 2019; Sanjari Moghaddam et al., 2019; Tang et al., 2021). Free Water (FW) is another marker sensitive to subtle damages to the axonal structure and to myelin membrane surrounding WM fibers, which has been linked to impaired processing speed (Duering et al., 2018), decreased episodic memory and executive function (Maillard et al., 2019), as well as with generally worse cognitive functions and with an increased risk for AD (Angoff et al., 2022; Ofori et al., 2019). Furthermore, using tractography on DTI data, WM fiber bundles might be reconstructed and disruption of structural connectivity between proximal and distant brain regions might be identified (Shenton et al., 2012). Changes in the properties of WM fiber tract connections (structural connectivity) are also important markers of WM integrity previously studied in obesity (Tohyama et al., 2020) and often associated to cognitive decline (Alfaro et al., 2018).

References

Alfaro, F.J., Gavrieli, A., Saade-Lemus, P., Lioutas, V.A., Upadhyay, J., Novak, V., 2018. White matter microstructure and cognitive decline in metabolic syndrome: a review of diffusion tensor imaging. Metabolism. https://doi.org/10.1016/j.metabol.2017.08.009

Angoff, R., Himali, J.J., Maillard, P., Aparicio, H.J., Vasan, R.S., Seshadri, S., Beiser, A.S., Tsao, C.W., 2022. Relations of Metabolic Health and Obesity to Brain Aging in Young to Middle-Aged Adults. J Am Heart Assoc 11. https://doi.org/10.1161/JAHA.121.022107

Ashburner, J., Friston, K.J., 2000. Voxel-Based Morphometry—The Methods. Neuroimage 11, 805–821. https://doi.org/10.1006/NIMG.2000.0582

Den Ruijter, H.M., Peters, S.A.E., Anderson, T.J., Britton, A.R., Dekker, J.M., Eijkemans, M.J., Engström, G., Evans, G.W., de Graaf, J., Grobbee, D.E., Hedblad, B., Hofman, A., Holewijn, S., Ikeda, A., Kavousi, M., Kitagawa, K., Kitamura, A., Koffijberg, H., Lonn, E.M., Lorenz, M.W., Mathiesen, E.B., Nijpels, G., Okazaki, S., O’Leary, D.H., Polak, J.F., Price, J.F., Robertson, C., Rembold, C.M., Rosvall, M., Rundek, T., Salonen, J.T., Sitzer, M., Stehouwer, C.D.A., Witteman, J.C., Moons, K.G., Bots, M.L., 2012. Common Carotid Intima-Media Thickness Measurements in Cardiovascular Risk Prediction. JAMA 308, 796. https://doi.org/10.1001/jama.2012.9630

Duering, M., Finsterwalder, S., Baykara, E., Tuladhar, A.M., Gesierich, B., Konieczny, M.J., Malik, R., Franzmeier, N., Ewers, M., Jouvent, E., Biessels, G.J., Schmidt, R., de Leeuw, F., Pasternak, O., Dichgans, M., 2018. Free water determines diffusion alterations and clinical status in cerebral small vessel disease. Alzheimer’s & Dementia 14, 764–774. https://doi.org/10.1016/j.jalz.2017.12.007

Fischl, B., Dale, A.M., 2000. Measuring the thickness of the human cerebral cortex from magnetic resonance images. Proc Natl Acad Sci U S A 97, 11050–11055. https://doi.org/10.1073/PNAS.200033797

Good, C.D., Scahill, R.I., Fox, N.C., Ashburner, J., Friston, K.J., Chan, D., Crum, W.R., Rossor, M.N., Frackowiak, R.S.J., 2002. Automatic Differentiation of Anatomical Patterns in the Human Brain: Validation with Studies of Degenerative Dementias. Neuroimage 17, 29–46. https://doi.org/10.1006/nimg.2002.1202

Grellmann, C., Bitzer, S., Neumann, J., Westlye, L.T., Andreassen, O.A., Villringer, A., Horstmann, A., 2015. Comparison of variants of canonical correlation analysis and partial least squares for combined analysis of MRI and genetic data. Neuroimage 107, 289–310. https://doi.org/10.1016/j.neuroimage.2014.12.025

Han, Y.P., Tang, X., Han, M., Yang, J., Cardoso, M.A., Zhou, J., Simó, R., 2021. Relationship between obesity and structural brain abnormality: Accumulated evidence from observational studies. Ageing Res Rev. https://doi.org/10.1016/j.arr.2021.101445

Jenkinson, M., Beckmann, C.F., Behrens, T.E.J., Woolrich, M.W., Smith, S.M., 2012. FSL. Neuroimage 62, 782–790. https://doi.org/10.1016/j.neuroimage.2011.09.015

Klein, A., Tourville, J., 2012. 101 Labeled Brain Images and a Consistent Human Cortical Labeling Protocol. Front Neurosci 6. https://doi.org/10.3389/fnins.2012.00171

KUBICKI, M., MCCARLEY, R., WESTIN, C., PARK, H., MAIER, S., KIKINIS, R., JOLESZ, F., SHENTON, M., 2007. A review of diffusion tensor imaging studies in schizophrenia. J Psychiatr Res 41, 15–30. https://doi.org/10.1016/j.jpsychires.2005.05.005

Lamar, M., Boots, E.A., Arfanakis, K., Barnes, L.L., Schneider, J.A., 2020. Common Brain Structural Alterations Associated with Cardiovascular Disease Risk Factors and Alzheimer’s Dementia: Future Directions and Implications. Neuropsychol Rev 30, 546–557. https://doi.org/10.1007/s11065-020-09460-6

Lee, N.R., Adeyemi, E.I., Lin, A., Clasen, L.S., Lalonde, F.M., Condon, E., Driver, D.I., Shaw, P., Gogtay, N., Raznahan, A., Giedd, J.N., 2016. Dissociations in Cortical Morphometry in Youth with Down Syndrome: Evidence for Reduced Surface Area but Increased Thickness. Cerebral Cortex 26, 2982–2990. https://doi.org/10.1093/cercor/bhv107

Lyttelton, O., Boucher, M., Robbins, S., Evans, A., 2007. An unbiased iterative group registration template for cortical surface analysis. Neuroimage 34, 1535–1544. https://doi.org/10.1016/j.neuroimage.2006.10.041

Ma, L.-Z., Huang, Y.-Y., Wang, Z.-T., Li, J.-Q., Hou, X.-H., Shen, X.-N., Ou, Y.-N., Dong, Q., Tan, L., Yu, J.-T., Initiative, A.D.N., 2019. Metabolically healthy obesity reduces the risk of Alzheimer’s disease in elders: a longitudinal study. Aging 11, 10939–10951. https://doi.org/10.18632/aging.102496

Maillard, P., Fletcher, E., Singh, B., Martinez, O., Johnson, D.K., Olichney, J.M., Farias, S.T., DeCarli, C., 2019. Cerebral white matter free water. Neurology 92. https://doi.org/10.1212/WNL.0000000000007449

Maingault, S., Tzourio-Mazoyer, N., Mazoyer, B., Crivello, F., 2016. Regional correlations between cortical thickness and surface area asymmetries: A surface-based morphometry study of 250 adults. Neuropsychologia 93, 350–364. https://doi.org/10.1016/j.neuropsychologia.2016.03.025

Medic, N., Ziauddeen, H., Ersche, K.D., Farooqi, I.S., Bullmore, E.T., Nathan, P.J., Ronan, L., Fletcher, P.C., 2016. Increased body mass index is associated with specific regional alterations in brain structure. Int J Obes 40, 1177–1182. https://doi.org/10.1038/ijo.2016.42

Meyer, M., Liem, F., Hirsiger, S., Jancke, L., Hanggi, J., 2014. Cortical Surface Area and Cortical Thickness Demonstrate Differential Structural Asymmetry in Auditory-Related Areas of the Human Cortex. Cerebral Cortex 24, 2541–2552. https://doi.org/10.1093/cercor/bht094

Ofori, E., DeKosky, S.T., Febo, M., Colon-Perez, L., Chakrabarty, P., Duara, R., Adjouadi, M., Golde, T.E., Vaillancourt, D.E., 2019. Free-water imaging of the hippocampus is a sensitive marker of Alzheimer’s disease. Neuroimage Clin 24, 101985. https://doi.org/10.1016/j.nicl.2019.101985

Panizzon, M.S., Fennema-Notestine, C., Eyler, L.T., Jernigan, T.L., Prom-Wormley, E., Neale, M., Jacobson, K., Lyons, M.J., Grant, M.D., Franz, C.E., Xian, H., Tsuang, M., Fischl, B., Seidman, L., Dale, A., Kremen, W.S., 2009. Distinct Genetic Influences on Cortical Surface Area and Cortical Thickness. Cerebral Cortex 19, 2728–2735. https://doi.org/10.1093/cercor/bhp026

Rakic, P., 1995a. A small step for the cell, a giant leap for mankind: a hypothesis of neocortical expansion during evolution. Trends Neurosci 18, 383–388. https://doi.org/10.1016/0166-2236(95)93934-P

Rakic, P., 1995b. A small step for the cell, a giant leap for mankind: a hypothesis of neocortical expansion during evolution. Trends Neurosci 18, 383–388. https://doi.org/10.1016/0166-2236(95)93934-P

Roy, B., Ehlert, L., Mullur, R., Freeby, M.J., Woo, M.A., Kumar, R., Choi, S., 2020. Regional Brain Gray Matter Changes in Patients with Type 2 Diabetes Mellitus. Sci Rep 10, 9925. https://doi.org/10.1038/s41598-020-67022-5

Sanjari Moghaddam, H., Ghazi Sherbaf, F., Aarabi, M.H., 2019. Brain microstructural abnormalities in type 2 diabetes mellitus: A systematic review of diffusion tensor imaging studies. Front Neuroendocrinol 55, 100782. https://doi.org/10.1016/j.yfrne.2019.100782

Schnack, H.G., van Haren, N.E.M., Brouwer, R.M., Evans, A., Durston, S., Boomsma, D.I., Kahn, R.S., Hulshoff Pol, H.E., 2015. Changes in Thickness and Surface Area of the Human Cortex and Their Relationship with Intelligence. Cerebral Cortex 25, 1608–1617. https://doi.org/10.1093/cercor/bht357

Shenton, M.E., Hamoda, H.M., Schneiderman, J.S., Bouix, S., Pasternak, O., Rathi, Y., Vu, M.A., Purohit, M.P., Helmer, K., Koerte, I., Lin, A.P., Westin, C.F., Kikinis, R., Kubicki, M., Stern, R.A., Zafonte, R., 2012. A review of magnetic resonance imaging and diffusion tensor imaging findings in mild traumatic brain injury. Brain Imaging Behav. https://doi.org/10.1007/s11682-012-9156-5

Tang, Q., Li, S., Yang, Z., Wu, M., Guo, Y., Yin, C., 2021. A narrative review of multimodal imaging of white matter lesions in type-2 diabetes mellitus. Ann Palliat Med 10, 12867–12876. https://doi.org/10.21037/apm-21-3299

Tohyama, S., Walker, M.R., Sammartino, F., Krishna, V., Hodaie, M., 2020. The Utility of Diffusion Tensor Imaging in Neuromodulation: Moving Beyond Conventional Magnetic Resonance Imaging. Neuromodulation: Technology at the Neural Interface 23, 427–435. https://doi.org/10.1111/ner.13107

Vuoksimaa, E., Panizzon, M.S., Chen, C.-H., Fiecas, M., Eyler, L.T., Fennema-Notestine, C., Hagler, D.J., Franz, C.E., Jak, A.J., Lyons, M.J., Neale, M.C., Rinker, D.A., Thompson, W.K., Tsuang, M.T., Dale, A.M., Kremen, W.S., 2016. Is bigger always better? The importance of cortical configuration with respect to cognitive ability. Neuroimage 129, 356–366. https://doi.org/10.1016/j.neuroimage.2016.01.049

Winkler, A.M., Greve, D.N., Bjuland, K.J., Nichols, T.E., Sabuncu, M.R., Håberg, A.K., Skranes, J., Rimol, L.M., 2018. Joint Analysis of Cortical Area and Thickness as a Replacement for the Analysis of the Volume of the Cerebral Cortex. Cereb Cortex 28, 738–749. https://doi.org/10.1093/cercor/bhx308

Worker, A., Blain, C., Jarosz, J., Chaudhuri, K.R., Barker, G.J., Williams, S.C.R., Brown, R., Leigh, P.N., Simmons, A., 2014. Cortical Thickness, Surface Area and Volume Measures in Parkinson’s Disease, Multiple System Atrophy and Progressive Supranuclear Palsy. PLoS One 9, e114167. https://doi.org/10.1371/journal.pone.0114167

Wright, I.C., McGuire, P.K., Poline, J.B., Travere, J.M., Murray, R.M., Frith, C.D., Frackowiak, R.S.J., Friston, K.J., 1995. A voxel-based method for the statistical analysis of gray and white matter density applied to schizophrenia. Neuroimage 2, 244–252. https://doi.org/10.1006/NIMG.1995.1032

[2] I. C. Wright *et al.*, ‘A voxel-based method for the statistical analysis of gray and white matter density applied to schizophrenia’, *Neuroimage*, vol. 2, no. 4, pp. 244–252, 1995, doi: 10.1006/NIMG.1995.1032.

[3] C. D. Good *et al.*, ‘Automatic Differentiation of Anatomical Patterns in the Human Brain: Validation with Studies of Degenerative Dementias’, *Neuroimage*, vol. 17, no. 1, pp. 29–46, Sep. 2002, doi: 10.1006/nimg.2002.1202.

[4] B. Roy *et al.*, ‘Regional Brain Gray Matter Changes in Patients with Type 2 Diabetes Mellitus’, *Sci Rep*, vol. 10, no. 1, p. 9925, Jun. 2020, doi: 10.1038/s41598-020-67022-5.

[5] C. Grellmann *et al.*, ‘Comparison of variants of canonical correlation analysis and partial least squares for combined analysis of MRI and genetic data’, *Neuroimage*, vol. 107, pp. 289–310, Feb. 2015, doi: 10.1016/j.neuroimage.2014.12.025.

[6] S. Maingault, N. Tzourio-Mazoyer, B. Mazoyer, and F. Crivello, ‘Regional correlations between cortical thickness and surface area asymmetries: A surface-based morphometry study of 250 adults’, *Neuropsychologia*, vol. 93, pp. 350–364, Dec. 2016, doi: 10.1016/j.neuropsychologia.2016.03.025.

[7] O. Lyttelton, M. Boucher, S. Robbins, and A. Evans, ‘An unbiased iterative group registration template for cortical surface analysis’, *Neuroimage*, vol. 34, no. 4, pp. 1535–1544, Feb. 2007, doi: 10.1016/j.neuroimage.2006.10.041.

[8] P. Rakic, ‘A small step for the cell, a giant leap for mankind: a hypothesis of neocortical expansion during evolution’, *Trends Neurosci*, vol. 18, no. 9, pp. 383–388, 1995, doi: 10.1016/0166-2236(95)93934-P.

[9] B. Fischl and A. M. Dale, ‘Measuring the thickness of the human cerebral cortex from magnetic resonance images’, *Proc Natl Acad Sci U S A*, vol. 97, no. 20, pp. 11050–11055, Sep. 2000, doi: 10.1073/PNAS.200033797.

[10] A. Klein and J. Tourville, ‘101 Labeled Brain Images and a Consistent Human Cortical Labeling Protocol’, *Front Neurosci*, vol. 6, 2012, doi: 10.3389/fnins.2012.00171.

[11] N. Medic *et al.*, ‘Increased body mass index is associated with specific regional alterations in brain structure’, *Int J Obes*, vol. 40, no. 7, pp. 1177–1182, Jul. 2016, doi: 10.1038/ijo.2016.42.

[12] P. Rakic, ‘A small step for the cell, a giant leap for mankind: a hypothesis of neocortical expansion during evolution’, *Trends Neurosci*, vol. 18, no. 9, pp. 383–388, Sep. 1995, doi: 10.1016/0166-2236(95)93934-P.

[13] M. S. Panizzon *et al.*, ‘Distinct Genetic Influences on Cortical Surface Area and Cortical Thickness’, *Cerebral Cortex*, vol. 19, no. 11, pp. 2728–2735, Nov. 2009, doi: 10.1093/cercor/bhp026.

[14] H. G. Schnack *et al.*, ‘Changes in Thickness and Surface Area of the Human Cortex and Their Relationship with Intelligence’, *Cerebral Cortex*, vol. 25, no. 6, pp. 1608–1617, Jun. 2015, doi: 10.1093/cercor/bht357.

[15] E. Vuoksimaa *et al.*, ‘Is bigger always better? The importance of cortical configuration with respect to cognitive ability’, *Neuroimage*, vol. 129, pp. 356–366, Apr. 2016, doi: 10.1016/j.neuroimage.2016.01.049.

[16] N. R. Lee *et al.*, ‘Dissociations in Cortical Morphometry in Youth with Down Syndrome: Evidence for Reduced Surface Area but Increased Thickness’, *Cerebral Cortex*, vol. 26, no. 7, pp. 2982–2990, Jul. 2016, doi: 10.1093/cercor/bhv107.

[17] M. Meyer, F. Liem, S. Hirsiger, L. Jancke, and J. Hanggi, ‘Cortical Surface Area and Cortical Thickness Demonstrate Differential Structural Asymmetry in Auditory-Related Areas of the Human Cortex’, *Cerebral Cortex*, vol. 24, no. 10, pp. 2541–2552, Oct. 2014, doi: 10.1093/cercor/bht094.

[18] A. M. Winkler *et al.*, ‘Joint Analysis of Cortical Area and Thickness as a Replacement for the Analysis of the Volume of the Cerebral Cortex.’, *Cereb Cortex*, vol. 28, no. 2, pp. 738–749, Feb. 2018, doi: 10.1093/cercor/bhx308.

[19] A. Worker *et al.*, ‘Cortical Thickness, Surface Area and Volume Measures in Parkinson’s Disease, Multiple System Atrophy and Progressive Supranuclear Palsy’, *PLoS One*, vol. 9, no. 12, p. e114167, Dec. 2014, doi: 10.1371/journal.pone.0114167.

[20] M. Jenkinson, C. F. Beckmann, T. E. J. Behrens, M. W. Woolrich, and S. M. Smith, ‘FSL’, *Neuroimage*, vol. 62, no. 2, pp. 782–790, Aug. 2012, doi: 10.1016/j.neuroimage.2011.09.015.

[21] M. E. Shenton *et al.*, ‘A review of magnetic resonance imaging and diffusion tensor imaging findings in mild traumatic brain injury’, 2012, *Springer Science and Business Media, LLC*. doi: 10.1007/s11682-012-9156-5.

[22] H. Sanjari Moghaddam, F. Ghazi Sherbaf, and M. H. Aarabi, ‘Brain microstructural abnormalities in type 2 diabetes mellitus: A systematic review of diffusion tensor imaging studies’, *Front Neuroendocrinol*, vol. 55, p. 100782, Oct. 2019, doi: 10.1016/j.yfrne.2019.100782.

[23] M. KUBICKI *et al.*, ‘A review of diffusion tensor imaging studies in schizophrenia’, *J Psychiatr Res*, vol. 41, no. 1–2, pp. 15–30, Jan. 2007, doi: 10.1016/j.jpsychires.2005.05.005.

[24] M. Lamar, E. A. Boots, K. Arfanakis, L. L. Barnes, and J. A. Schneider, ‘Common Brain Structural Alterations Associated with Cardiovascular Disease Risk Factors and Alzheimer’s Dementia: Future Directions and Implications’, *Neuropsychol Rev*, vol. 30, no. 4, pp. 546–557, Dec. 2020, doi: 10.1007/s11065-020-09460-6.

[25] Y. P. Han *et al.*, ‘Relationship between obesity and structural brain abnormality: Accumulated evidence from observational studies’, Nov. 01, 2021, *Elsevier Ireland Ltd*. doi: 10.1016/j.arr.2021.101445.

[26] Q. Tang, S. Li, Z. Yang, M. Wu, Y. Guo, and C. Yin, ‘A narrative review of multimodal imaging of white matter lesions in type-2 diabetes mellitus’, *Ann Palliat Med*, vol. 10, no. 12, pp. 12867–12876, Dec. 2021, doi: 10.21037/apm-21-3299.

[27] L.-Z. Ma *et al.*, ‘Metabolically healthy obesity reduces the risk of Alzheimer’s disease in elders: a longitudinal study’, *Aging*, vol. 11, no. 23, pp. 10939–10951, Dec. 2019, doi: 10.18632/aging.102496.

[28] H. M. Den Ruijter *et al.*, ‘Common Carotid Intima-Media Thickness Measurements in Cardiovascular Risk Prediction’, *JAMA*, vol. 308, no. 8, p. 796, Aug. 2012, doi: 10.1001/jama.2012.9630.

[29] M. Duering *et al.*, ‘Free water determines diffusion alterations and clinical status in cerebral small vessel disease’, *Alzheimer’s & Dementia*, vol. 14, no. 6, pp. 764–774, Jun. 2018, doi: 10.1016/j.jalz.2017.12.007.

[30] P. Maillard *et al.*, ‘Cerebral white matter free water’, *Neurology*, vol. 92, no. 19, May 2019, doi: 10.1212/WNL.0000000000007449.

[31] E. Ofori *et al.*, ‘Free-water imaging of the hippocampus is a sensitive marker of Alzheimer’s disease’, *Neuroimage Clin*, vol. 24, p. 101985, 2019, doi: 10.1016/j.nicl.2019.101985.

[32] R. Angoff *et al.*, ‘Relations of Metabolic Health and Obesity to Brain Aging in Young to Middle-Aged Adults’, *J Am Heart Assoc*, vol. 11, no. 6, Mar. 2022, doi: 10.1161/JAHA.121.022107.

[33] S. Tohyama, M. R. Walker, F. Sammartino, V. Krishna, and M. Hodaie, ‘The Utility of Diffusion Tensor Imaging in Neuromodulation: Moving Beyond Conventional Magnetic Resonance Imaging’, *Neuromodulation: Technology at the Neural Interface*, vol. 23, no. 4, pp. 427–435, Jun. 2020, doi: 10.1111/ner.13107.

[34] F. J. Alfaro, A. Gavrieli, P. Saade-Lemus, V. A. Lioutas, J. Upadhyay, and V. Novak, ‘White matter microstructure and cognitive decline in metabolic syndrome: a review of diffusion tensor imaging’, Jan. 01, 2018, *W.B. Saunders*. doi: 10.1016/j.metabol.2017.08.009.
